# Computation of Oxidation Potentials of Solvated Nucleobases by Static and Dynamic Multilayer Approaches

**DOI:** 10.1021/acs.jcim.2c00234

**Published:** 2022-06-30

**Authors:** Jesús Lucia-Tamudo, Gustavo Cárdenas, Nuria Anguita-Ortiz, Sergio Díaz-Tendero, Juan J. Nogueira

**Affiliations:** † Department of Chemistry, Universidad Autónoma de Madrid, 28049 Madrid, Spain; ‡ Institute for Advanced Research in Chemistry (IAdChem), Universidad Autónoma de Madrid, 28049 Madrid, Spain; ¶ Condensed Matter Physics Center (IFIMAC), Universidad Autónoma de Madrid, 28049 Madrid, Spain

## Abstract

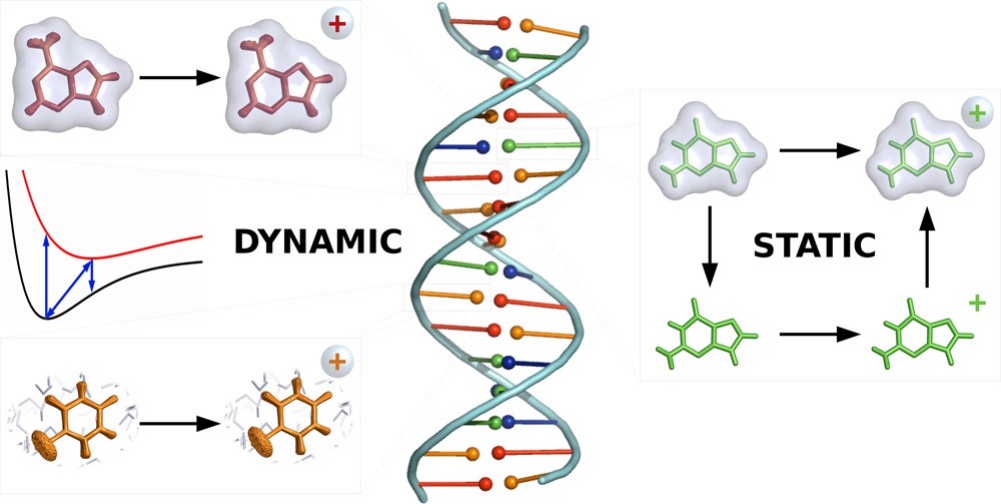

The determination of the redox properties of nucleobases is of paramount importance to get insight into the charge-transfer processes in which they are involved, such as those occurring in DNA-inspired biosensors. Although many theoretical and experimental studies have been conducted, the value of the one-electron oxidation potentials of nucleobases is not well-defined. Moreover, the most appropriate theoretical protocol to model the redox properties has not been established yet. In this work, we have implemented and evaluated different static and dynamic approaches to compute the one-electron oxidation potentials of solvated nucleobases. In the static framework, two thermodynamic cycles have been tested to assess their accuracy against the direct determination of oxidation potentials from the adiabatic ionization energies. Then, the introduction of vibrational sampling, the effect of implicit and explicit solvation models, and the application of the Marcus theory have been analyzed through dynamic methods. The results revealed that the static direct determination provides more accurate results than thermodynamic cycles. Moreover, the effect of sampling has not shown to be relevant, and the results are improved within the dynamic framework when the Marcus theory is applied, especially in explicit solvent, with respect to the direct approach. Finally, the presence of different tautomers in water does not affect significantly the one-electron oxidation potentials.

## 1 Introduction

**Figure 1:**
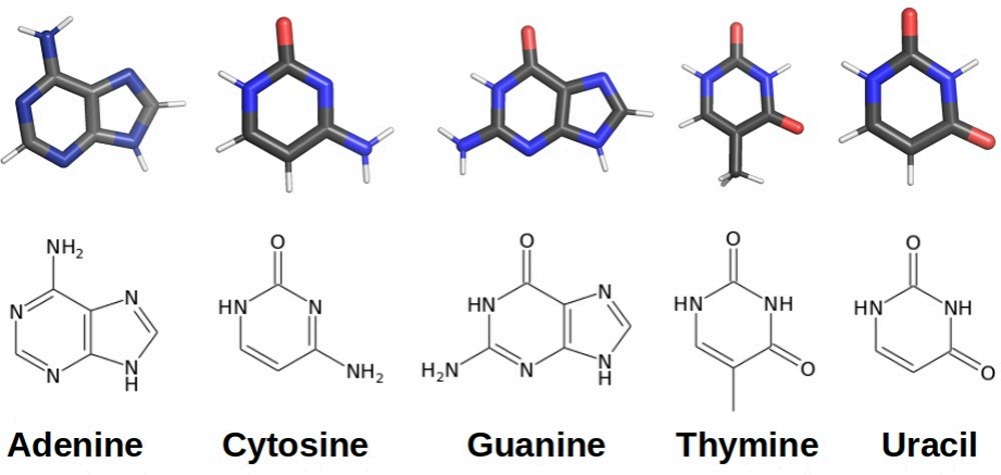
Schematic representation of the five nucleobases.

The determination of the one-electron redox potentials of the nucleobases by experimental measurements is a very difficult task due to the irreversibility of the process and the low solubility of some nucleobases in water.^1−6^ In the same way, the computation of the potentials is also challenging due to the several factors that can be considered in the theoretical model by means of different approaches, such as vibrational sampling, solvent effects, and the level of theory at which the electronic structure of the nucleobases (see Figure 1) are described. However, an accurate characterization of the redox properties of nucleobases is crucial to understand the molecular mechanism of different charge-transfer processes in which the DNA constituents are involved, such as those occurring in biosensor devices.^7,8^ DNA-based biosensors have been proven to be a convenient choice when trying to detect specific sequences of nucleic acids due to the DNA capability of hybridization.^9−13^ Recently, these devices have also been employed as nanowires or for the detection of organic analytes and heavy metals.^9,14−16^ In order to carry out the detection task, charge-transfer processes often play an important role, and the nucleobase moiety has been shown to be the main constituent of the nucleotides that is involved in these phenomena.^1^ Thus, the attainment of the redox properties of nucleobases is fundamental to get insight into the functioning mechanism of DNA-based biosensors.

This work is aimed to establish the most appropriate computational strategy to evaluate the one-electron oxidation potential of the five nucleobases present in DNA and RNA, by combining different theoretical models, and to compare the results with the data available in the literature. We will use the term “one-electron oxidation potential” as the potential of the oxidation process but considered in the direction of a reduction reaction. Thus, the “one-electron oxidation potential” is a reduction potential as it is usually considered in the literature by convention. Many different experiments have been performed during the last decades, leading to a large variety of results. For example, Faraggi and co-workers employed cyclic voltammetry, differential pulse polarography, and pulse radiolysis to determine the oxidation potentials at different pH values.^3^ Jovanovic and Simic employed pulse radiolysis to obtain the oxidation potentials at pH = 13.0.^4^ Jovanovic also conducted further experiments with Steenken and determined the oxidation potential for guanine using kinetic rate measurements at physiological pH.^17,18^ Analogously, Steenken also obtained these values for the rest of nucleobases, which were supported by Burrows and co-workers.^19,20^ Seidel and co-workers performed fluorescence quenching experiments to determine the potentials in acetonitrile^5^ and compared them with those reported in aqueous solution by Kittler and co-workers at pH = 6.5.^21^ As can be seen in Table 1, all these experimental studies have provided a large range of one-electron oxidation potentials for the five nucleobases.

**Table 1:** Experimental^3−5,17−23^ and Theoretical^1,2,24−31^ Ranges Provided by the Literature for One-Electron Oxidation Potentials of the Nucleobases in Aqueous Solution

nucleobase	experiment (V)	theory (V)
adenine	1.20–1.63	1.38–2.22
cytosine	1.44–1.86	1.76–2.50
guanine	0.80–1.53	1.10–1.88
thymine	1.29–1.73	1.42–2.46
uracil	1.34–1.75	1.62–2.57

**Figure 2:**
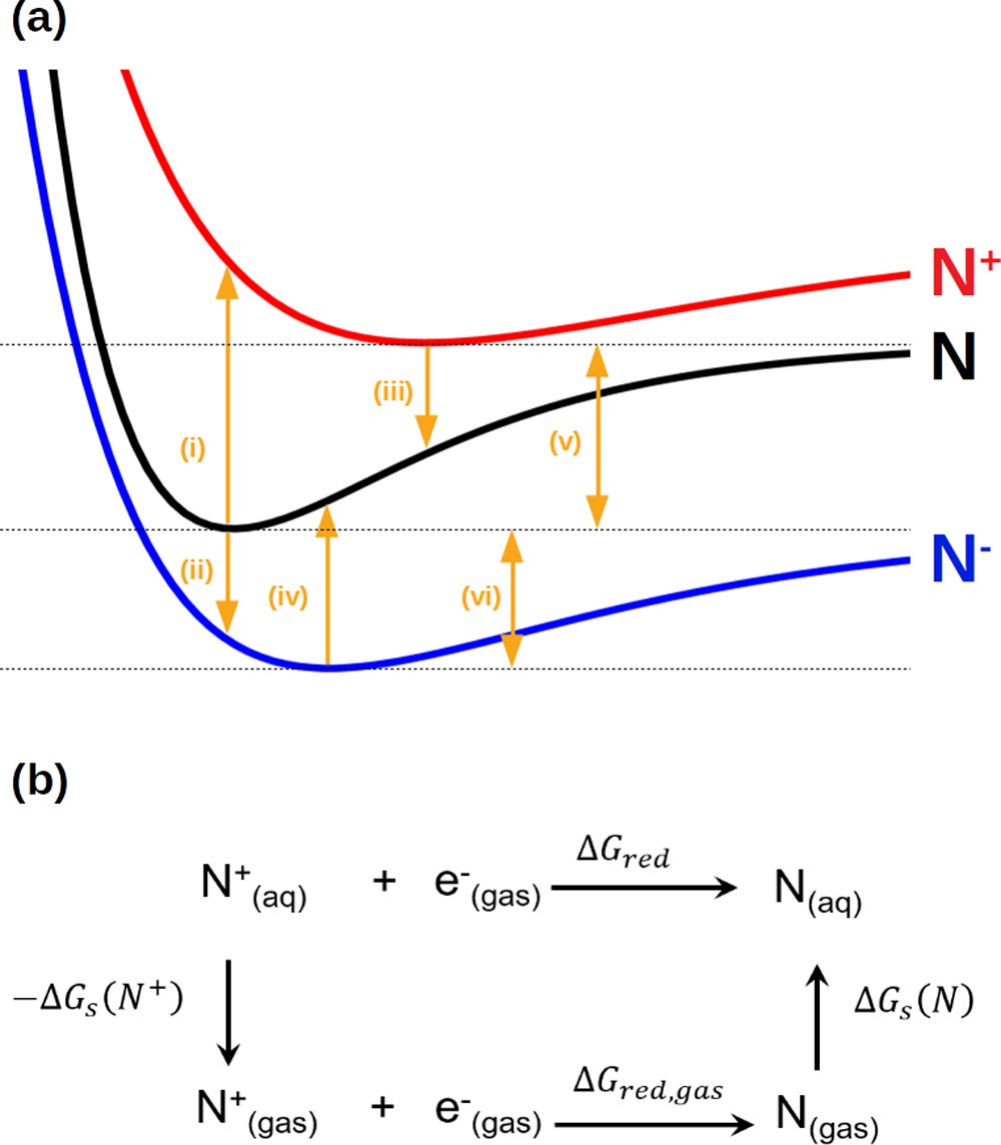
(a) Schematic representation of the different energy terms related to reduction and oxidation processes: (i) vertical ionization energy (VIE), (ii) vertical electron affinity (VEA), (iii) vertical attachment energy (VAE), (iv) vertical detachment energy (VDE), (v) adiabatic ionization energy (AIE), and (vi) adiabatic electron affinity (AEA). (b) Thermodynamic cycle to compute reduction free energy in solvent, Δ*G*_red_, from the solvation free energies of the oxidized species, Δ*G*_s_(N^+^), reduced species, Δ*G*_s_(N), and electron, Δ*G*_s_(e^–^), and the reduction free energy in the gas phase Δ*G*_red,gas_.

From the computational perspective, many studies based on static and dynamic approaches have also been developed with the aim of computing the redox properties of nucleobases. For example, within a static picture, Baik et al. determined the one-electron oxidation potentials for the nucleobases from the adiabatic ionization energies (AIEs, see Figure 2a) computed by density functional theory (DFT) combined with a continuum-solvent approach.^25^ Thapa and Schlegel^27^ simulated the methyl-substituted nucleobases and some of their tautomers using also a DFT/continuum-solvent approach and taking into account the contribution of the formation of the solvated electron. Crespo-Hernández and co-workers^32^ evaluated if there was a correlation between the calculated vertical ionization energy (VIE) or the vertical electron affinity (VEA) using DFT/continuum models and the redox potentials of 20 organic molecules, for which the experimental redox potentials were well-known in acetonitrile. After obtaining a linear correlation, they used such a relation to obtain the redox properties of nucleobases in acetonitrile through the calculated VIEs and VEAs. On the other hand, Li et al. designed a protocol to investigate aromatic compounds, including the nucleobases,^24^ which consists of a thermodynamic cycle and computations based on DFT and continuum solvent models. In such a protocol, solvation and structural relaxation effects were determined separately. Paukku and Hill^28^ and Lewis et al.^29^ determined the redox potentials of nucleobases based on the same thermodynamic cycle but using a different level of theory and continuum solvation models. They also compared the VIEs with the AIEs, and the VEAs with the adiabatic electron affinities (AEAs), properties intimately related to the oxidation and reduction potentials (see Figure 2a). Psciuk and co-workers^2^ computed the redox potentials of methyl-substituted nucleobases using the same strategy, but they took into account the different tautomers of the nucleobases that can be present in a protic solvent such as water.

The one-electron oxidation potentials of nucleobases have also been computed based on dynamic approaches, specifically, using classical molecular dynamics (MD)^33,34^ or quantum mechanics/molecular mechanics MD^35^ (QM/MM MD) simulations. In this context, Wang and co-workers^30^ evolved classical MD trajectories for all the nucleobases in order to obtain an appropriate ensemble of conformational configurations of the molecules in aqueous phase using the TIP3P^36^ force field for the description of water. Then, from different snapshots, QM/MM MD simulations were performed in order to relax the nucleobase geometries, and finally the VIEs, VEAs, and vertical detachment energies (VDEs) were obtained by DFT/MM calculations, from which the redox potentials were determined. Zhang et al.^26^ followed a similar methodology, but they used QM/MM MD simulations to relax the neutral, cationic, and anionic species starting from snapshots taken from a classical MD simulation of the neutral species. Then, VIEs, VEAs, AIEs, AEAs, and VDEs were computed by means of a DFT/MM approach. All these energetic terms are schematically represented in Figure 2a. Mukherjee and co-workers reported the electron affinity of uracil applying a QM/MM approach, where the QM region was described at the EOM-CCSD level of theory.^31^ Recently, D’Annibale et al.^1^ reported the redox properties of nucleobases and nucleosides using an innovative methodology. In a first step, they conducted classical MD simulations to obtain a full ensemble of geometries of the neutral and cationic forms of the nucleobases in aqueous solvation. Then, in a second step, they performed DFT/MM calculations using the perturbed matrix method.^37,38^ Finally, they applied Marcus theory to estimate the one-electron oxidation potentials using the VIEs and the vertical attachment energies (VAEs). As in the case of the experimental measurements, the computed oxidation potentials listed in Table 1 lie in a large range of values. Moreover, they are considerably larger than the experimental potentials. Thus, a clear value for the one-electron oxidation potentials of the nucleobases cannot be extracted from the literature.

In spite of all the previously mentioned efforts, it is still not clear which methodology is more appropriate to compute the redox potentials for the nucleobases and, in general, for organic molecules. In fact, the use of thermodynamic cycles has been recently compared with the direct determination of the reduction potentials from the AIEs for different molecules, and a general conclusion could not be drawn.^39^ In addition, the calculation of redox properties in solvent or in biological media also requires an accurate description of the environment in terms of both interactions and sampling. In this work, we have computed the one-electron oxidation potentials of the five nucleobases in water using different protocols and theoretical models within static and dynamic frameworks aimed to different goals: (i) to compare the use of two different thermodynamic cycles with the direct determination of the oxidation potentials from the AIEs within a static framework; (ii) to evaluate the introduction of sampling effects and compare two different dynamic protocols where the potentials are computed from the AIEs and by the Marcus theory; (iii) to investigate the effect of solvent models using implicit (COSMO) and explicit (TIP3P) solvation; (iv) to assess the accuracy of different DFT functionals for each of the static and dynamic protocols; (v) to evaluate the importance of considering different tautomeric species.

## 2 Methods

### 2.1 Static Approaches

The reduction free energy of the process shown in Figure 2b can be written as the free energy difference between the solvated reduced and oxidized species as
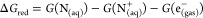
1where the free energy of each individual species can be written as the summation of the electronic energy, *E*_e_, and the thermal correction to the Gibbs free energy, *G*_T_, which includes electronic, translational, rotational, and vibrational contributions. Both energy terms are usually computed by using a continuum solvent approach, such as the polarizable continuum model (PCM)^40−45^ or the conductor-like screening model (COSMO).^46,47^ Thus, eq 1 can be written as
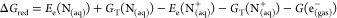
2The term *G*_c_(e^–^) is the free energy of the electron in the gas phase. The fact that the electron is considered in the gas phase in the half reaction is just a formalism. The electron contribution cancels out when the second half reaction is considered.^48^ Therefore, it is irrelevant which formalism is employed while it is the same for both half reactions. The reduction potential, *E*_red_, is related to the free energy as follows:
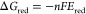
3where *F* is the Faraday constant and *n* is the number of exchanged electrons. Typically, reduction potentials are given with respect to a reference potential. A common choice as reference is the standard hydrogen electrode (SHE), whose reduction potential is *E*_red,SHE_^0^ = 4.281 V, used in previous works.^48−52^ Although this value is a common choice when COSMO is employed to describe solvent effects, it is not clear which value is consistent with this solvent model.^53^ It is important to highlight here that the value of 4.281 V of the SHE was obtained using the Fermi–Dirac statistics to account for the free energy of the electron, whose value is −0.867 kcal/mol.^54−56^ Therefore, it is necessary to cancel this electron contribution by explicitly including the free energy of the electron in the computation of the reaction free energy for the half reaction under investigation, for example, through eq 1. Thus, the reduction potential relative to the SHE value reads as
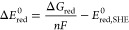
4

The use of eqs 1–4 to obtain the reduction potentials is termed here as static direct calculation. In this methodology, the terms *E*_e_ and *G*_T_ for the reduced and oxidized species in eq 2 are computed at their corresponding optimized geometries in a continuum solvent model. In other words, the free energy of the reduction process is obtained from the AIE (see Figure 2a).

It has been recently stated that the use of the static direct recipe can lead to errors in the determination of the free energy.^39^ Specifically, the calculation of the thermal correction *G*_T_ in a continuum solvent is not completely correct. First, continuum models have been parametrized so that the electronic energy *E*_e_ of the solute including the interaction with the solvent reproduces the experimental solvation energy. Therefore, thermal effects are already included implicitly in *E*_e_ and the computation of *G*_T_ in the continuum solvent model double counts these effects. Second, the ideal-gas partition functions employed when computing the thermal corrections for the solvated species are not correct in solvent. Consequently, it has been suggested that the use of a thermodynamic cycle, where the thermal corrections are computed in the gas phase, is more accurate.

The free energy of the reduction reaction can also be expressed by using the thermodynamic cycle shown in Figure 2b as
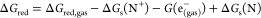
5where Δ*G*_s_(*I*) is the solvation free energy of the species *I* and Δ*G*_red,gas_ is the free energy of the reduction process in the gas phase. The last one can be written as the summation of electronic *E*_e_ and thermal correction *G*_T_ contributions:
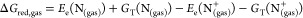
6In addition, the solvation free energy, Δ*G*_s_(*I*), is calculated as the difference between the electronic energy of the solute in solvent and in the gas phase:
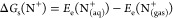
7
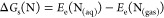
8By using the above-described thermodynamic cycle, the thermal corrections are computed in the gas phase though the terms *G*_T_(N_(gas)_) and *G*_T_(N_(gas)_^+^) in eq 6. Since the partition functions are derived from the ideal gas, rigid rotor, and harmonic oscillator approaches, their application should be more appropriate in the gas phase than in aqueous phase, although they could also introduce important errors when, for example, anharmonic motions are present in the system. In addition, corrections to the thermal energy due to the discrepancies between the solvent phase and the gas phase are considered in the computation of the electronic energies through eqs 7 and 8.

In this work, two different versions of the same thermodynamic cycle have been tested. Their main difference lies in the way in which the solvation free energies are calculated. In the first one (static cycle 1), the solvation free energies Δ*G*_s_(N) and Δ*G*_s_(N^+^) are evaluated with the geometries of the reduced and oxidized species optimized in solvent and in the gas phase, that is, the geometry relaxation of the solute upon solvation is considered. In the second version (static cycle 2), the solvation free energies of eqs 7 and 8 are computed by employing the relaxed geometry in the gas phase. This second approximation does not take into account the relaxation of the solute in solvent, but it is still often found in the literature because it avoids the geometry optimization in continuum solvation models, where convergence is sometimes difficult to achieve.^29,32^

### 2.2 Dynamic Approaches

The reduction potentials can also be determined by using dynamic methodologies where conformational motion (sampling) is introduced in the theoretical model. As a first approach, eq 2 can be applied to obtain the free energy of the oxidation process. If the thermal correction is not explicitly computed, contrary to the static protocols, but it is accounted in an indirect and approximate way by considering an ensemble of geometries over which the potential energy is averaged, eq 2 can be rewritten as
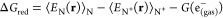
9where the subscripts of the angle brackets indicate the phase space where the potential energy average is computed. Thus, the ensemble average of the potential energy of the reduced species N in the first term of eq 9 is computed in the phase space of N, while the ensemble average of the potential energy of the oxidized species N^+^ in the second term of eq 9 is computed in the phase space of N^+^. The ensemble averages for each of the species can be easily determined by running classical MD simulations for each phase space, selecting several snapshots, and computing the potential energy for the selected snapshots by QM/MM or QM/continuum approaches, where the solute is included in the QM region and the solvent is described by a MM force field or by a continuum model. Finally, the average of the potential energies for each ensemble is introduced in eq 9 and the free energy is obtained. We will refer to this methodology as dynamic direct explicit or dynamic direct implicit approach, depending on the solvent model employed in the potential-energy calculations.

**Figure 3:**
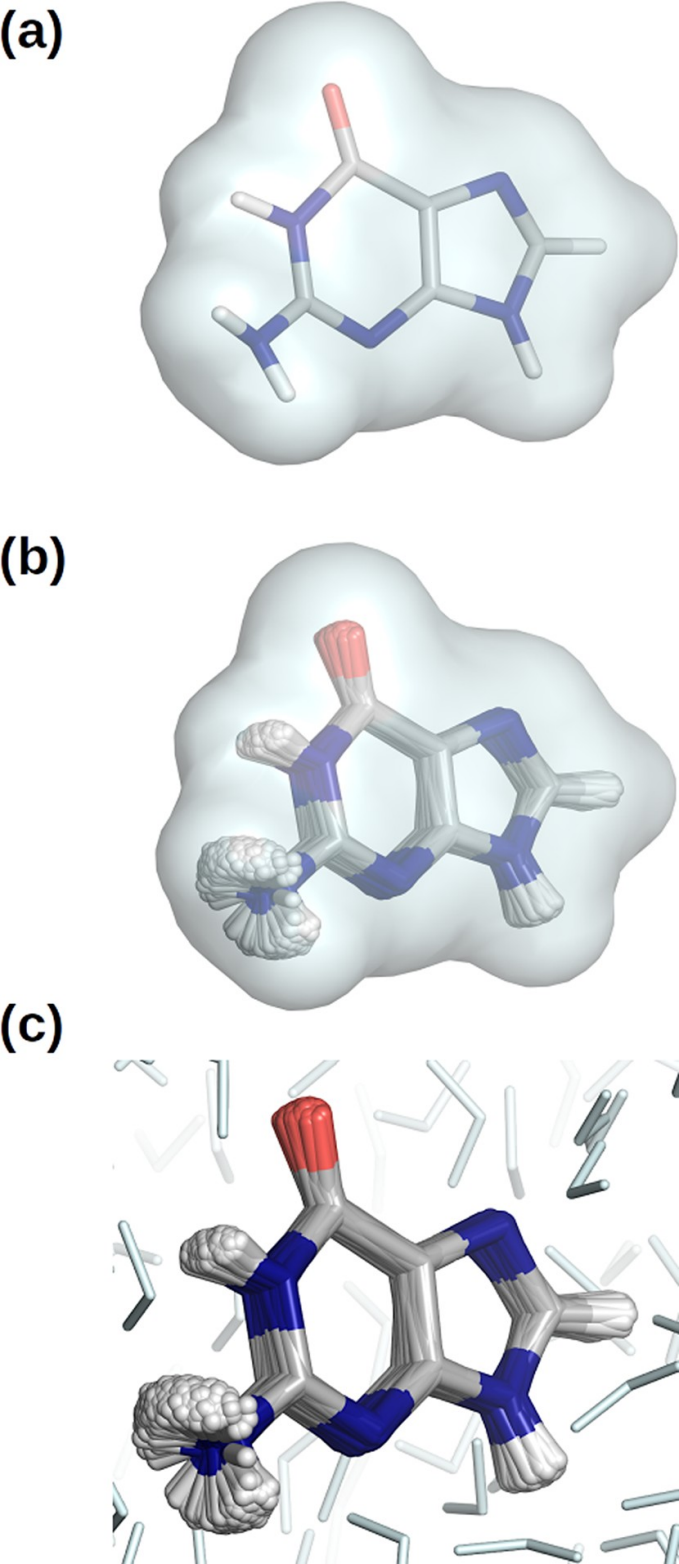
Representation of the different types of calculations performed. (a) Static calculations in which the solvent is described by the continuum COSMO solvation model. (b) Dynamic calculations in which the sampling of the solute is taking into account and the solvent is described by COSMO as in panel a. (c) Dynamic calculations in which the sampling of the solute and solvent is taking into account since the solvent is described explicitly with TIP3P. Several frames have been aligned to represent the vibrational motion of the solute in panels b and c. Color code: N in blue, C in gray, O in red, H in white, and solvent (water) in light blue.

Reduction potentials can also be computed by using the Marcus theory, where sampling and environmental effects are included. It can be shown that the free energy (or the Helmholtz energy at constant volume) can be expressed as follows:^57,58^
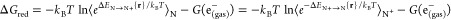
10where Δ*E*_N→N^+^_{**r**} and Δ*E*_N^+^→N_{**r**} are the VIE and the VAE, respectively, computed on the appropriate ensemble of N or N^+^. Assuming that the solvent response is linear with respect to a change in the solute, Marcus theory can be applied.^59−65^ Hence, both the distributions of VIEs and VAEs will be Gaussian functions and their standard deviations will be the same. Under these circumstances eq 10 can be simplified to
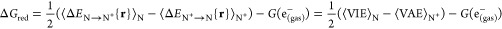
11In order to compute ⟨VIE⟩_N_ (⟨VAE⟩_N^+^_), classical MD simulations are run for the reduced species N (oxidized species N^+^) in its own phase space. Then, several snapshots are chosen along the dynamics, and for those selected snapshots the VIEs (VAEs) are calculated by QM/MM or QM/continuum approaches, where only the solute is included in the QM region (see Figure 3). Finally, the VIE (VAE) values are averaged. Depending on the solvent model employed in the VIE (VAE) computations, this methodology will be named dynamic Marcus explicit or dynamic Marcus implicit approach.

In order to assess the viability of the Marcus theory within these systems, three conditions must be fulfilled: (i) the distributions of the VIE and VAE must show a Gaussian shape, (ii) the standard deviations (σ_VIE_, σ_VAE_) must be the same, and (iii) the reorganization energy λ, defined in eq 12, must be equal to the reorganization energies of both the neutral species and the cation, defined in eqs 13 and 14.^66^
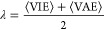
12
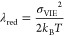
13
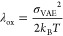
14

Under these circumstances the Marcus theory can be successfully used, while in any other case the Marcus theory suffers deviations. To overcome these problems, the quadratic model developed by Matyushov and Voth^67,68^ can be employed. Thus, eq 15 is based on a correction that solves the problems associated with nonlinear solvation response using a three-parameter model in contrast with the two-parameter model typically used in Marcus theory.^66^
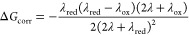
15

## 3 Computational Details

### 3.1 Static Calculations

All the QM, QM/continuum, and QM/MM calculations were performed using the NWChem package.^69^ Three different functionals were tested within the static and dynamic methodologies: PBEOP,^70−72^ which provided previously accurate results for nucleobases,^73^ and the B3LYP^74−77^ and M06-2X^78^ functionals, widely employed in the computation of ground-state properties. The selected basis set was 6-311G(d).^79,80^

All the nucleobases were optimized in their neutral and cationic forms in both gas phase and aqueous phase using the three functionals and the basis set mentioned above. The optimization of the cationic form of cytosine did not converge for B3LYP and M06-2X. In these cases, the optimized geometries of the neutral and cationic cytosine using PBEOP were used in order to compute the energy of the system with B3LYP and M06-2X. The aqueous solvent in the QM/continuum calculations was described by COSMO.^46,47^ A frequency calculation was performed for each geometry to ensure that an energy minimum was reached and to compute the zero-point energy, which is part of the thermal correction. By calculating the energy of the cationic and neutral species both in the gas phase and in solvent, the oxidation potentials for the three different models described above are obtained: static direct, static cycle 1, and static cycle 2 approaches.

### 3.2 Dynamic Calculations

Classical MD simulations were run with the AMBER20 package,^81^ and the systems were built up with AmberTools 20^81^ and different homemade scripts. For both the cation and the neutral forms of each nucleobase, three different sets of force field parameters were developed, based on quantum mechanics calculations performed with the PBEOP, B3LYP, and M06-2X functionals by the following procedure. First of all, the Hessian matrix for the optimized geometries obtained in the static calculations in aqueous phase was computed. Bond and bond angle parameters for the nucleobases were obtained from the Hessian matrix by the Seminario method using each of the considered functionals.^82^ Parameters for dihedral angles, improper torsions, and Lennard–Jones nonbonded terms were taken from the generalized Amber force field (GAFF).^83^ Electrostatic potential (ESP) charges were obtained from a DFT calculation in aqueous phase. Each nucleobase was solvated in a tetragonal simulation box of around (60 × 57 × 61) Å^3^ with approximately 17000 water molecules, which were described with the TIP3P solvation model.^36^ For the cationic form, a chloride anion was also added to neutralize the system, described by the Joung and Cheatham parameters.^84^

After the setup of the different systems, the same dynamic protocol was followed for all of them. First, the system was minimized for 10000 steps in which the steepest descent algorithm^85^ was used for the first 5000 steps and the Newton–Raphson algorithm^86^ was used for the last 5000 steps. After that, a constant volume (NVT) progressive heating to 300 K was performed for 500 ps. The Langevin thermostat was applied to control the temperature with a collision frequency of 2 ps^–1^. Then, an additional 500 ps simulation was run at 300 K in the NVT ensemble. Afterward, a 1 ns simulation was run in the NPT ensemble to equilibrate the volume of the system and reach the correct density. Finally, a 500 ns production simulation was run in the NPT ensemble. The Berendsen barostat with isotropic position scaling and a pressure relaxation time of 2 ps was employed to maintain the pressure constant at 1 bar. During the full protocol, the particle-mesh Ewald method^87^ with a grid spacing of 1.0 Å was used to compute the electrostatic interactions, and a 10 Å cutoff for the nonbonded interactions was chosen. The SHAKE algorithm^88−90^ restrained the bonds involving hydrogen atoms, and a time step of 2 fs was used during the heating, equilibration, and production stages.

For each cationic and neutral trajectory of the five nucleobases, 200 snapshots were fetched randomly from the last 450 ns of the production trajectories. In the case of the snapshots of the neutral species, the VIEs were computed by running electrostatic embedding QM/MM calculations, where the solvent was described by TIP3P, and polarizable-embedding QM/COSMO calculations. For the QM/COSMO calculations, the explicit solvent molecules were removed from the different snapshots and replaced by COSMO. Regarding the cationic trajectories, the VAEs were computed also by QM/MM and QM/COSMO calculations in the same way. These calculations were run for the three functionals and basis set described above for the static approaches using NWChem.^69^ Since the optimization of the cationic cytosine molecule did not converge for B3LYP and M06-2X and, therefore, the Hessian matrix could not be obtained, the MD simulations of neutral and cationic cytosine with the force field parameters obtained from PBEOP were used in order to compute the energy of the system with B3LYP and M06-2X. All these calculations were combined as explained in the previous section to obtain the one-electron oxidation potentials for the dynamic direct implicit, dynamic direct explicit, dynamic Marcus implicit, and dynamic Marcus explicit approaches.

## 4 Results

### 4.1 Static Protocols

**Figure 4:**
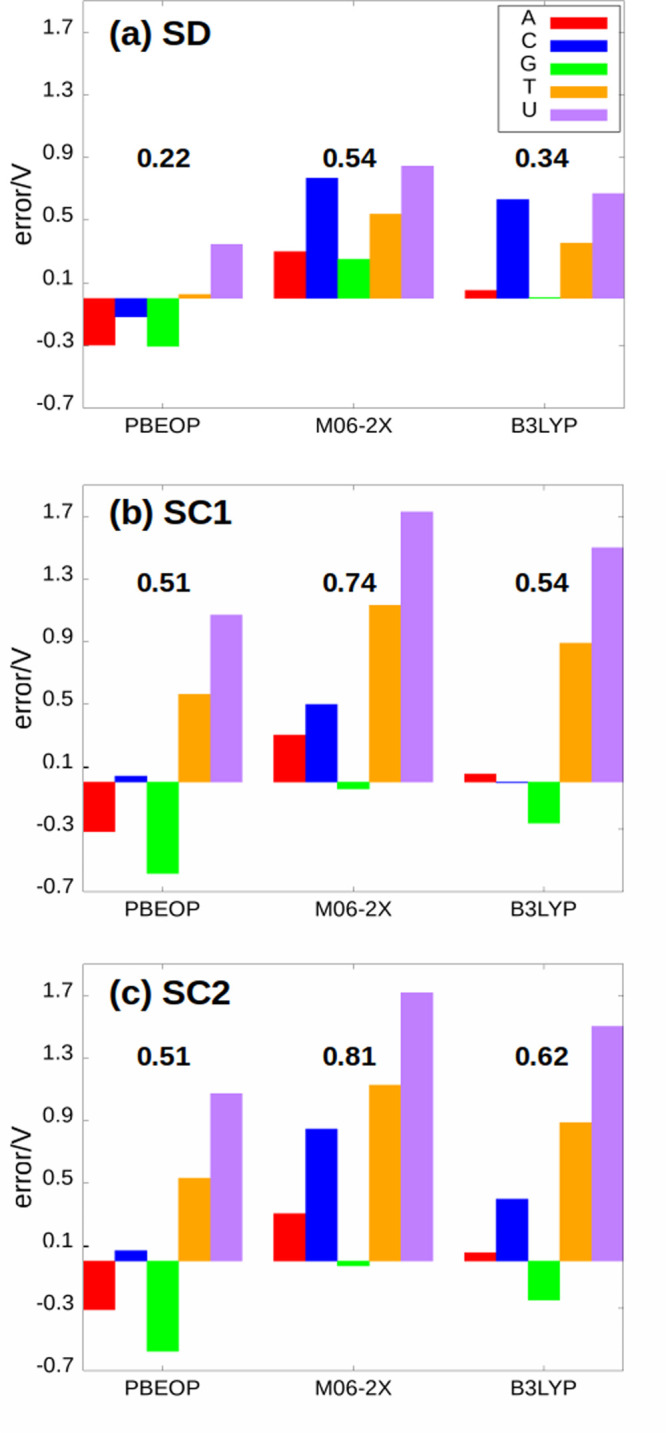
Errors of the calculated one-electron oxidation potentials for the five nucleobases obtained with the PBEOP, M06-2X, and B3LYP functionals with respect to the reference experimental oxidation potentials using the three static protocols described in the text: (a) static direct (SD), (b) static cycle 1 (SC1), and (c) static cycle 2 (SC2). The values next to the bars are the MUEs for each functional. Color code: adenine (A) in red, cytosine (C) in blue, guanine (G) in green, thymine (T) in orange and uracil (U) in purple.

We start the discussion by comparing the results from the three different stationary approaches described above: static direct, static cycle 1, and static cycle 2. The error of these three protocols for each of the five nucleobases employing the PBEOP, M06-2X, and B3LYP functionals with respect to experimental measurements is shown in Figure 4. Specifically, due to the broad range of experimental values found in the literature, the unsigned error for each nucleobase is estimated as the absolute value of the difference between the computed oxidation potential and the average value of the experimental range listed in the second column of Table 1. It should be noted that the comparison with the experimental data must be done with caution due to the broad variety of experimental results found in the literature. At first glance, comparison with higher-level electronic-structure calculations could seem a better choice. However, our goal is to assess not only the accuracy of different DFT functionals but also the accuracy of different solvent models and static and dynamic protocols. Therefore, to choose a gold standard theoretical model is a very challenging task, and this is the reason why the quality of the different protocols will be discussed in terms of the errors computed with respect to the experimental values. In general, when comparing the results given by the different DFT functionals for all the nucleobases, PBEOP provides the most consistent potentials with respect to the literature with mean unsigned errors (MUEs) of 0.22, 0.51, and 0.51 V for the static direct, static cycle 1, and static cycle 2 approaches, respectively. Moreover, the B3LYP and M06-2X functionals overestimate the potentials for all nucleobases except guanine, with MUEs lying within 0.34–0.62 V and 0.54–0.81 V for B3LYP and M06-2X, respectively. If Figure 4 is examined in more detail, one finds that the accuracy of the functionals is system dependent: the potential for adenine computed with B3LYP presents the lowest error for the three static protocols, the potential for guanine is more accurate when calculated with M06-2X, and for the pyrimidine nucleobases, cytosine, thymine, and uracil, PBEOP is the most appropriate functional.

When we compare the three protocols, the static direct scheme is the one that provides the closest values with respect to the experiments for the three functionals (see Figure 4). This means that either the inaccuracies introduced in the thermal correction to the free energy when a continuum solvation model is used in the static direct protocol, as previously discussed,^39^ are not important for the nucleobases or there exists error cancellation with other approximations inherent to the theoretical model employed. A more careful inspection of Figure 4 reveals that the determination of the one-electron oxidation potential for adenine seems to be invariant with respect to the theoretical protocol. In the case of cytosine, the static cycle 1 performs the best, although the static direct calculation with PBEOP provides a reasonable result with an error of ∼0.3 V. The thermodynamic cycles completely fail when describing the oxidation potentials of thymine and uracil, presenting errors of ∼0.9 V for thymine and ∼1.3 V for uracil. The static cycle 2 scheme provides slightly higher MUE values than the static cycle 1, especially for cytosine. This shows that geometry relaxation when going from vacuum to solvent is relevant for cytosine, while the use of the vacuum geometries in COSMO calculations does not introduce significant errors for the other nucleobases. Overall, the best static strategy to compute the one-electron oxidation potential for the nucleobases has been proven to be the static direct method with the PBEOP functional.

### 4.2 Dynamic Protocols

The one-electron oxidation potentials have also been computed within a dynamic approach, where an ensemble of 200 geometries for each nucleobase, selected from classical MD simulations, were considered. Different factors that may influence the accuracy of the computations within this dynamic framework will be discussed in the following sections. First, it is important to achieve convergence with respect to the number of selected snapshots. Second, although the classical trajectories for sampling have been evolved in explicit solvation, the calculation of the oxidation potentials has been carried out in explicit and implicit models by using electrostatic embedding QM/MM and polarizable embedding QM/continuum schemes, providing potentials with different accuracy and convergence behavior. Third, the free energy of the oxidation process for the different nucleobases has been obtained by applying the direct approximation, as in the static scenario, and the more sophisticated Marcus theory. Finally, the role of tautomers, which can be relevant in protic solvents such as water, have also been analyzed.

#### 4.2.1 Convergence of the Calculations

**Figure 5:**
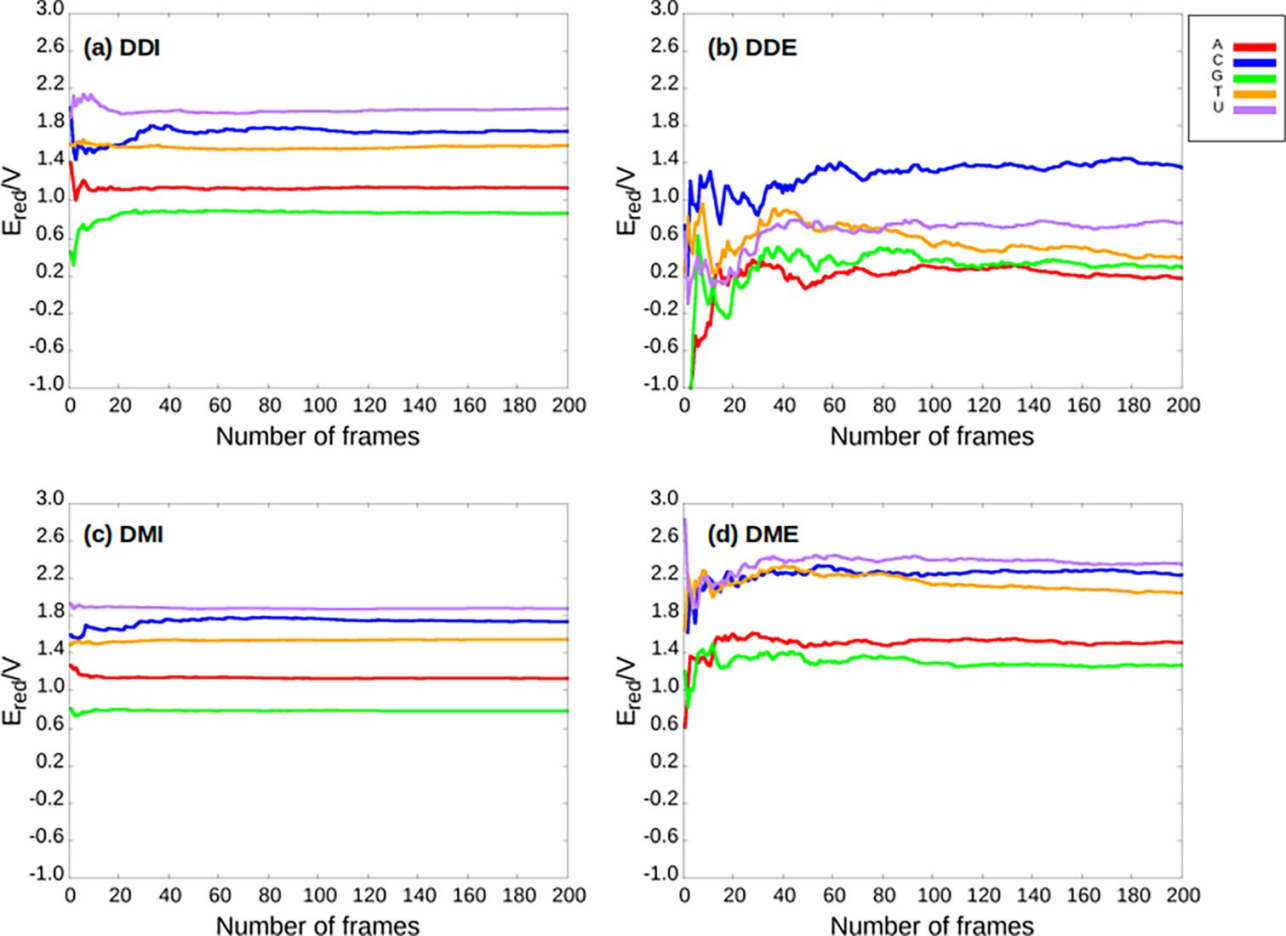
Evolution of the average one-electron oxidation potentials computed with PBEOP with respect to the number of frames for the five nucleobases using different dynamic protocols: (a) dynamic direct implicit (DDI), (b) dynamic direct explicit (DDE), (c) dynamic Marcus implicit (DMI), (d) dynamic Marcus explicit (DME). Color code: adenine (A) in red, cytosine (C) in blue, guanine (G) in green, thymine (T) in orange and uracil (U) in purple.

Figure 5 shows the evolution of the one-electron oxidation potentials with the number of snapshots considered for the five nucleobases computed by applying the direct and Marcus protocols with implicit COSMO and explicit TIP3P solvents. As can be seen, convergence is successfully reached for all the dynamic approaches when using 200 snapshots randomly fetched from the classical MD trajectories. However, the calculations performed with COSMO converge faster than those performed with TIP3P. Specifically, convergence is achieved for the continuum solvent after considering 40–60 snapshots, while it is necessary to average over 100 snapshots to converge the oxidation potentials for explicit solvation. In addition, the oscillations of the average potentials are smaller for COSMO than for TIP3P. This is consistent with the fact that in the implicit solvation model the different configurations of the solvent are averaged in every calculation, while the solvent configuration around the solute is different in each calculation with explicit solvent. Therefore, the variation of the oxidation potential along the different snapshots is larger in TIP3P because both the solute and solvent undergo important changes within the ensemble. In contrast, in the implicit calculations, only the solute geometry suffers important modifications, and the cavity that represents the solvent adapts to these modifications with small deformations of its shape and tesserae charges.

When comparing the dynamic direct with the dynamic Marcus approaches some differences can be observed (see Figure 5). First, convergence is reached with a smaller number of snapshots when applying Marcus theory, especially when using the explicit TIP3P model as solvent. In addition, the oscillations observed for the Marcus theory are smaller than those for the direct approach. This can be understood by realizing that the free energy of the oxidation process is computed as the average value of the VIE and VEA in the Marcus theory (see eq 11) and, thus, the number of single-point calculations employed in the dynamic Marcus protocols is double that in the dynamic direct approaches (800 vs 400 single-point calculations for each nucleobase). The difference in the convergence behavior between both dynamic protocols is almost negligible when using COSMO due to the averaging nature of the implicit model, as was explained above. In summary, the one-electron oxidation potential values converge for all the dynamic protocols when ∼100 snapshots are used. However, convergence is achieved faster by applying Marcus theory and using the continuum solvent.

#### 4.2.2 Effect of Sampling

The introduction of conformational sampling in the theoretical model may modify the properties of a system when compared with the static scenario.^91−94^ This arises from the fact that large sized systems do not present a single clear global minimum in their potential-energy surface but many local minima that are thermally accessible. As a result, static calculations only describe the properties of the system associated with one of these minima, whose selection is arbitrary. In these situations, there is a need to explore different conformations along the relevant regions of the potential-energy surface. This is achieved in the present work by running classical MD simulations.

**Figure 6:**
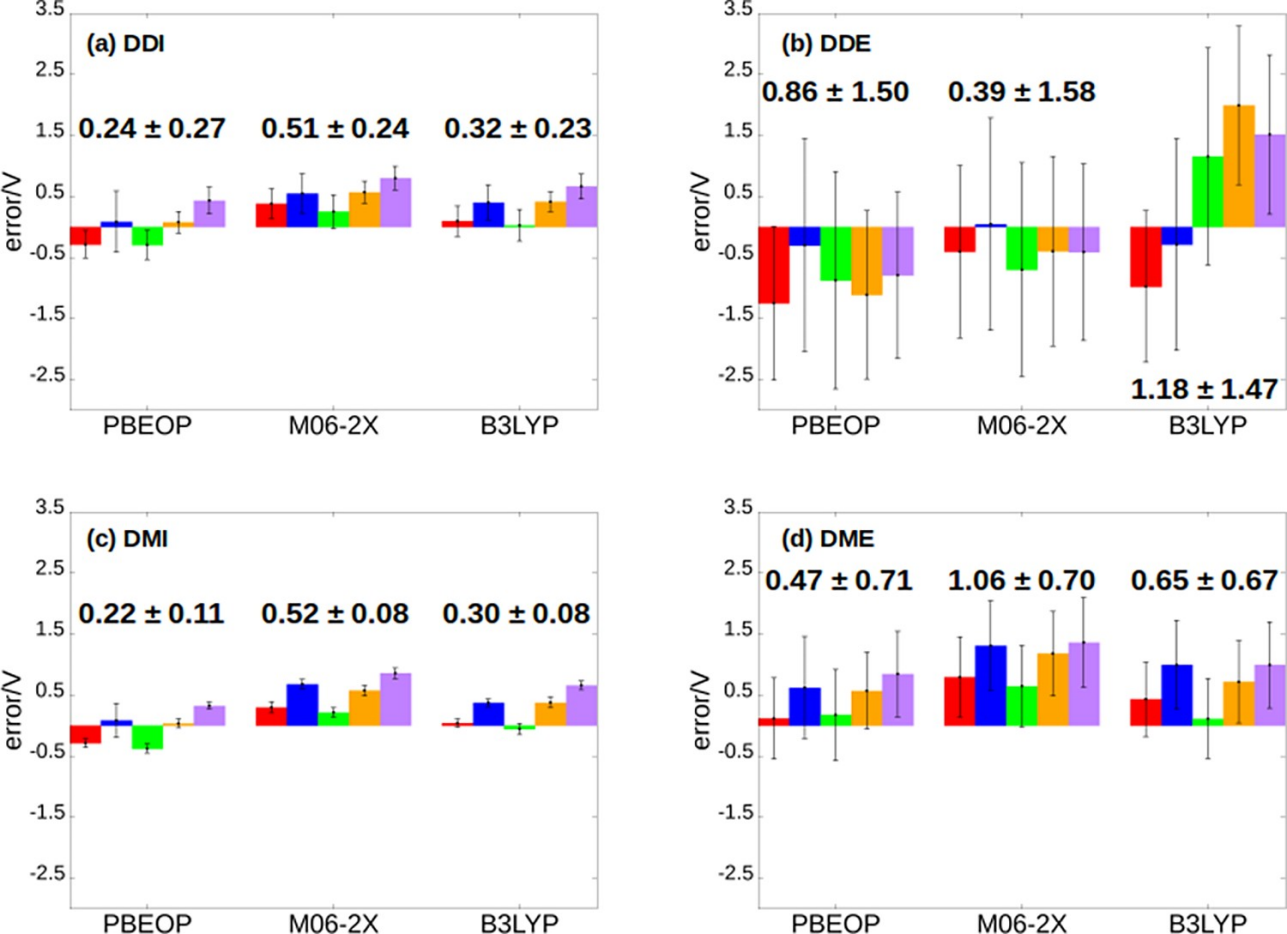
Errors of the calculated one-electron oxidation potentials for the five nucleobases obtained with the PBEOP, M06-2X, and B3LYP functionals with respect to the reference experimental oxidation potentials using the four dynamic protocols described in the text: (a) dynamic direct implicit (DDI), (b) dynamic direct explicit (DDE), (c) dynamic Marcus implicit (DMI), and (d) dynamic Marcus explicit (DME). Error bars represent the standard deviation of the calculated potentials, and the values next to the bars are the MUEs with their standard deviation for each functional. Color code: adenine (A) in red, cytosine (C) in blue, guanine (G) in green, thymine (T) in orange, and uracil (U) in purple.

In order to discuss the effect of sampling, the errors in the one-electron oxidation potentials obtained by the static direct protocol with COSMO, displayed in Figure 4a, will be compared with those obtained by the dynamic direct implicit approach, shown in Figure 6a. The MUEs for the static direct approach are 0.22, 0.54, and 0.34 V for PBEOP, M06-2X, and B3LYP, respectively, while they are 0.24, 0.51, and 0.32 V for the dynamic direct implicit approach. Therefore, static and dynamic oxidation potentials are very similar, and thus, sampling effects are not relevant in this situation. The nucleobase that presents the most important variation in its potential when going from the static to the dynamic approach is cytosine, whose oxidation potential difference between both approaches is ∼0.2 V. If the nucleobases are analyzed individually, the introduction of sampling decreases (increases) the error of pyrimidines (purines), although these variations, as already said, are not important.

#### 4.2.3 Effect of the Solvation Model

The presence of an environment, for example, solvent, can also induce modifications in the molecular properties.^92,95,96^ Moreover, the magnitude of these modifications may be different depending on the solvent model employed. In the present work, two different solvent models have been investigated (see Figure 3), namely, the implicit COSMO and explicit TIP3P models. The effect of the solvent description on the oxidation potentials can be seen by doing the following comparisons: dynamic direct implicit (Figure 6a) vs dynamic direct explicit (Figure 6b) protocols and dynamic Marcus implicit (Figure 6c) vs dynamic Marcus explicit (Figure 6d) protocols. For both comparisons, it can be seen that the MUEs of the calculations performed with the explicit solvent are clearly larger than those of the calculations performed with COSMO. The only exception is found for the dynamic direct protocols employing the M06-2X functional, for which the MUE is 0.51 V for COSMO and 0.39 V for TIP3P. Therefore, in general, the one-electron oxidation potentials obtained with the implicit solvent model are more accurate than those obtained with the explicit one. At first glance, this could seem surprising since it is usually stated that explicit models are more accurate than implicit ones because specific interactions, such as hydrogen bonding, are taken into account. However, the effect of explicit solvent molecules is often introduced by an electrostatic-embedding QM/MM scheme, as was performed here, where the solvent molecules (MM region) polarize the solute (QM region) but the solvent is not polarized by the solute. In the case of implicit models, solvent effects are introduced by polarizable-embedding QM/continuum schemes, where there exists a mutual polarization between solute and solvent, which, in principle, is more accurate than an electrostatic embedding. The fact that the COSMO calculations present a smaller error than the TIP3P ones indicates that, in the particular case of computing oxidation potentials for the nucleobases, polarization effects are more relevant than specific interactions, for example, hydrogen bonding. In addition, other factors could also be behind the better performance of COSMO, such as solvent reorganization, which is not considered in the TIP3P explicit model. Therefore, the use of the COSMO model to represent the water molecules is more appropriate than the use of TIP3P regarding the computation of oxidation potentials of nucleobases.

Another important difference found between the two solvent models is the variation of the oxidation potential along the snapshots of the ensemble. This is reflected in the standard deviations, which are represented in Figure 6 by the error bars. The standard deviations found in the explicit solvent calculations (0.42–1.58 V) are significantly larger than those for the implicit solvent calculations (0.08–0.27 V). As discussed above, this is explained by the nature of the implicit model, whose cavity represents an average situation of all possible solvent configurations. It is important to mention that, in principle, the larger standard deviation does not make TIP3P a worse model than COSMO. However, the large MUE values obtained with TIP3P demonstrate the worse performance of the explicit model.

#### 4.2.4 Viability of the Application of Marcus Theory

**Figure 7:**
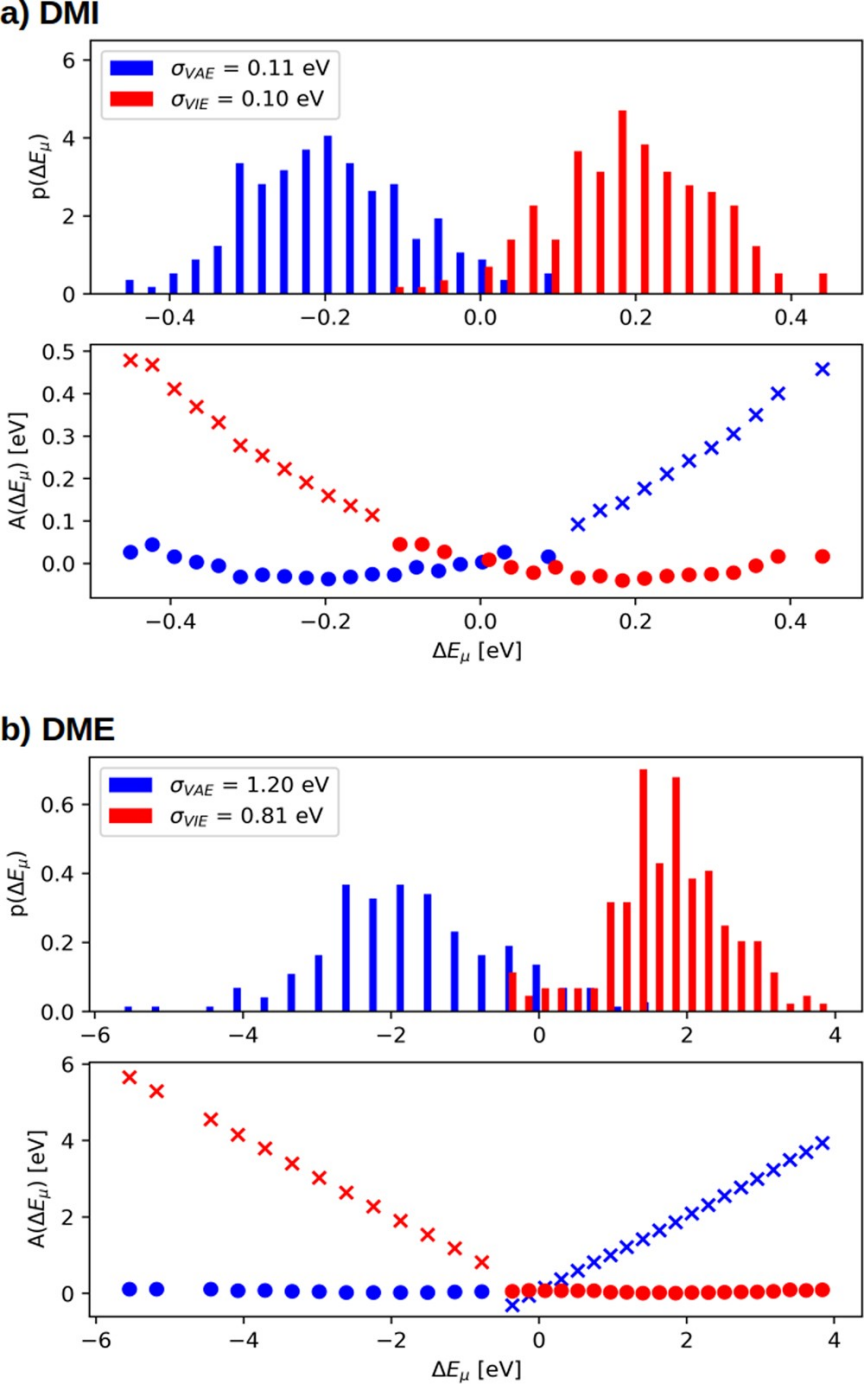
Distribution of VIE (red) and VAE (blue) for guanine when using (a) implicit or (b) explicit solvation models. Δ*E*_μ_ represents the VIE and VAE centered at the average value of ⟨VIE⟩ and ⟨VAE⟩. *p*(Δ*E*_μ_) is the relative population of each Δ*E*_μ_, while *A*(Δ*E*_μ_) is −*k*_B_*T* ln(*p*(Δ*E*_μ_)). Points are the values obtained from calculations, while the extrapolated crosses are determined using Δ*A*_red_(*E*_μ_) = Δ*A*_ox_(*E*_μ_) + Δ*E*_μ_.^97^

The implementation of Marcus theory is a well-known alternative for the computation of the one-electron oxidation potentials. However, the application of this formulation needs to be assessed since some features must be fulfilled. First of all, the distributions of both the VIE and the VEA must be Gaussian distribution functions. As shown in Figure 7 for guanine, the distributions of both dynamic Marcus implicit and explicit methodologies are close to be Gaussian shaped, but they are not perfect Gaussian functions; this indicates that it would be necessary to consider a much large number of geometries in the computation of the VIE and VAE, especially in the case of explicit solvation. The plots for the other nucleobases are not shown for simplicity because they are similar to the ones for guanine. Moreover, the consequence of having Gaussian functions is that the corresponding free energy curves should be intersecting parabolas, as it should be expected from Marcus theory. However, as seen in the lower panels of Figure 7a,b, this is again only partially fulfilled by the implicit solvation case. Second, the standard deviations of both VIE and VAE must be equal for a specific system, and as a consequence, the reorganization energies defined in eqs 12, 13 and 14 must also be the same. This requirement is accomplished in the dynamic Marcus implicit scheme but not in the explicit case, as can be observed in Table 2, where the values for the five nucleobases and the three functionals are listed.

**Table 2:** Calculated Standard Deviations of the VIEs, σ_VIE_, and the VAEs, σ_VAE_, for Each of the Functionals Employed in the Dynamic Marcus Implicit and Explicit Approaches^a^

	PBEOP				M06-2X				B3LYP			
	implicit		explicit		implicit		explicit		implicit		explicit	
	σ_VIE_	σ_VAE_	σ_VIE_	σ_VAE_	σ_VIE_	σ_VAE_	σ_VIE_	σ_VAE_	σ_VIE_	σ_VAE_	σ_VIE_	σ_VAE_
A	0.09	0.10	0.81	1.01	0.12	0.12	0.80	1.13	0.10	0.10	0.76	1.02
C	0.41	0.42	1.16	1.30	0.09	0.11	0.86	1.18	0.07	0.8	0.85	1.18
G	0.10	0.11	0.81	1.20	0.11	0.13	0.84	1.12	0.12	0.13	0.75	1.05
T	0.10	0.11	0.77	1.08	0.11	0.13	0.91	1.16	0.11	0.12	0.84	1.01
U	0.07	0.10	0.80	1.10	0.11	0.14	0.87	1.21	0.09	0.09	0.81	1.06

a All of the standard deviations are given in V.

All these results provide evidence that the implicit solvent calculations follow a Marcus regime, while important deviations from the Marcus theory, which should be corrected, are found for the explicit solvent model. This can be explained once more in terms of the averaging character of the COSMO solvation model, which requires the use of a smaller number of geometries. Therefore, the Marcus explicit scheme will be corrected by eq 15, while this is not necessary in the dynamic Marcus implicit situation.

#### 4.2.5 Direct vs Marcus Theory Protocols

The application of the Marcus theory to compute the one-electron oxidation potentials could be thought to provide more accurate results than the direct protocol due to the greater complexity of the Marcus formulation. However, this is the case only when using explicit solvation but not implicit. As can be seen in Figure 6a,c, the MUEs of the dynamic direct implicit approach are 0.24, 0.51, and 0.32 V for PBEOP, M06-2X, and B3LYP, respectively, while similar MUE values are obtained for the dynamic Marcus implicit protocol (0.22, 0.52, and 0.30 V). Therefore, the accuracy of both dynamic procedures is very similar when using COSMO to reproduce solvent effects. In addition, these MUEs are also similar to the ones displayed in Figure 6a for the static direct (implicit) approach (0.22, 0.54, and 0.34 V). As was discussed in a previous section, sampling effects are not relevant in this particular situation. These three protocols (static direct implicit, dynamic direct implicit, and dynamic Marcus implicit) present the best agreement with the experimental measurements, especially when the PBEOP functional is used to describe the electronic structure of the nucleobases.

The situation is different for the explicit solvation calculations. The dynamic Marcus approach (Figure 6d) agrees better with the experiment than the dynamic direct approach (Figure 6b) for the PBEOP and B3LYP functionals, and the opposite is true for the M06-2X functional. It is interesting to mention that the M06-2X functional also presents a smaller MUE in TIP3P (Figure 6b) than in COSMO (Figure 6a) for the dynamic direct protocol. Therefore, it seems that the results computed by M06-2X profit from error cancellation when this functional is combined with TIP3P in the dynamic direct approach. Nevertheless, despite the improvement of the results when applying Marcus theory with respect to the dynamic direct protocol in TIP3P for PBEOP and B3LYP, the most accurate results are still obtained for implicit solvation.

**Figure 8:**
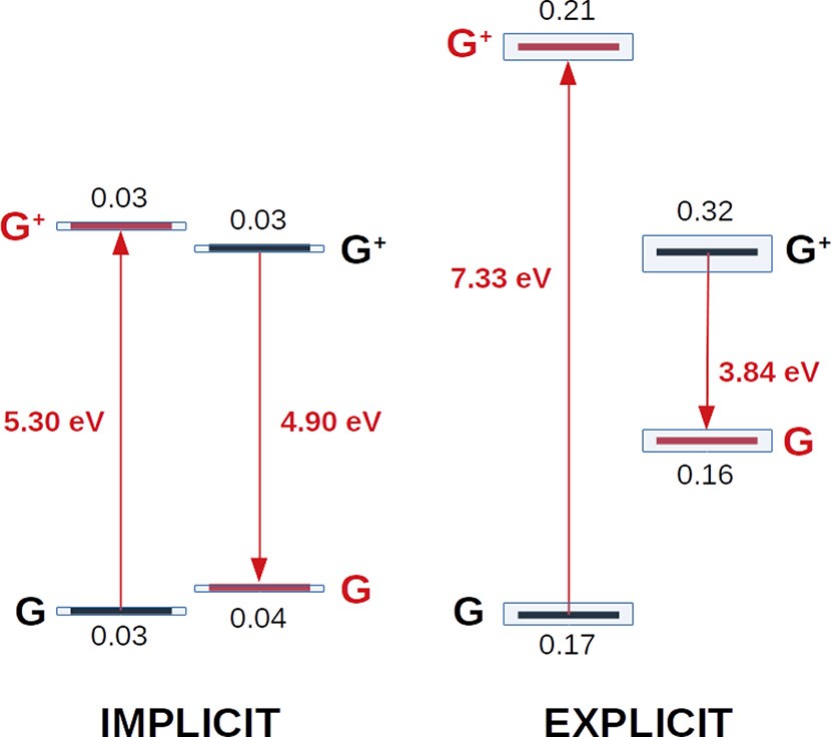
Schematic representation of the energy gaps employed in the dynamic direct and Marcus approaches using implicit and explicit solvation. The results shown were obtained for guanine with the PBEOP functional. Black labels (G and G^+^) represent geometrically relaxed species, while red labels (G and G^+^) account for species whose energy was computed without geometry relaxation. Arrows pointing up and down represent the VIE and VAE, respectively. Blue boxes and black numbers account for the standard deviation of the energy of the correspondent state. All values are in eV.

It is worth discussing some differences between the implicit and explicit solvent calculations within the dynamic framework. Figure 8 shows the different calculations that are involved in the dynamic direct and Marcus protocols. In these calculations, first, two different classical MD simulations are run for the neutral and the cationic nucleobases (represented by the black labels G and G^+^). In the case of the direct approach, the free energy of the oxidation process is obtained directly from the energy difference between the cationic and neutral trajectories. When the Marcus theory is instead applied, the VIE is computed from the neutral trajectory by removing an electron without relaxing the geometry, and the VAE is computed from the cationic trajectory by adding an electron without relaxing the geometry. These unrelaxed situations are represented in Figure 8 by the red labels G and G^+^. Finally, the oxidation free energy is computed as the average of the VIE and VAE. In the case of the explicit solvent calculations, the same water molecules present in the dynamics are employed in the QM/MM energy calculations, while in the case of the implicit solvent calculations the explicit solvent molecules from the dynamics are replaced by the COSMO model in the QM/continuum energy calculations.

As can be seen in Figure 8, the unrelaxed geometries are higher in energy than the relaxed ones, as expected. As a consequence, the VIE is always higher than the VAE (5.30 vs 4.90 eV in COSMO and 8.09 vs 4.08 eV in TIP3P). However, the energy difference between the VIE and VAE is significantly larger in TIP3P than in COSMO because the energy difference between the relaxed and unrelaxed trajectories is also larger in the explicit solvent. This can be rationalized as follows. When the VIE is computed by removing an electron from the snapshots of the neutral trajectory, in the COSMO solvent model the tesserae charges of the cavity change to adapt to the positive charge of the solute because of the existing mutual polarization between solute and solvent. Therefore, the reorganization of the solvent charges is stabilizing the unrelaxed geometry of the cationic nucleobase. As a result, the energy difference between the unrelaxed and the relaxed cationic solute is not too large. However, in the electrostatic-embedding QM/MM calculations with TIP3P, the charges of the solvent do not relax upon ionization of the neutral trajectory because TIP3P is a fixed-charge force field. Therefore, the energy of the unrelaxed cationic trajectory is much higher than the energy of the relaxed cationic trajectory. The same explanation holds for the VAE computation. When the cationic trajectory is neutralized, the unrelaxed neutral geometry is much more stabilized in COSMO than in TIP3P. Interestingly, despite this dissimilar behavior between the unrelaxed trajectories for COSMO and TIP3P, the results obtained by the dynamic Marcus protocol in both solvent models are not too different because TIP3P overestimates the VIEs but underestimates the VAEs, making the average of the two magnitudes similar to the one computed with COSMO. However, the slightly better performance of the COSMO calculations is likely explained by the polarizable nature of the solvent model.

Finally, Figure 8 also displays the standard deviations of the QM/TIP3P and QM/COSMO energy calculations for the relaxed and unrelaxed trajectories. As can be seen, the standard deviations for COSMO are much smaller (0.03–0.04 eV) than those for TIP3P (0.16–0.32 eV). These larger energy oscillations observed for the explicit solvent cause the large standard deviations in the oxidation potentials shown in Figure 6 for both the dynamic direct and dynamic Marcus protocols.

### 4.3 DFT Functionals

The electronic-structure calculations for the static and dynamic approaches described in this work were carried out using the PBEOP, M06-2X, and B3LYP functionals. A general feature observed in Figures 4 and 6 is the wide range of errors found for a given DFT functional. The use of a higher level of theory might provide more homogeneous results among the five nucleobases, but its application would be very computationally demanding for the dynamic protocols and the different solvent models and when dealing with larger systems. In general terms, PBEOP is the functional that presents the best agreement with the experimental oxidation potentials, as can be seen in Figures 4 and 6. In addition, PBEOP provides the lowest one-electron oxidation potentials for the five nucleobases, followed by B3LYP and then by M06-2X, as illustrated in Table 3. It is interesting to highlight that despite the M06-2X and B3LYP functionals have a hybrid nature, which implies a higher computational cost than GGA functionals (e.g., PBEOP), the results are more accurate when PBEOP is employed, supporting the choice of this functional for the computation of the redox properties of these systems. For the schemes that describe the solvent with a continuum solvation model, B3LYP and M06-2X give oxidation potentials ∼0.3 V and ∼0.5 V, respectively, greater than PBEOP for all nucleobases in both the static and dynamic protocols. These differences between PBEOP and B3LYP become a bit larger when the solvent is explicitly described by TIP3P in the dynamic Marcus protocol, whereas the errors with respect to PBEOP barely change for M06-2X. Specifically, the oxidation potentials computed with M06-2X and B3LYP are ∼0.5–0.7 V and ∼0.1–0.3 V larger, respectively, than those computed using PBEOP. The only situation where PBEOP does not perform the best is for the dynamic direct explicit protocol, for which M06-2X presents the lowest MUE value (0.39 V), followed by PBEOP (0.86 V) and B3LYP (1.18 V).

**Table 3:** Calculated One-Electron Oxidation Potentials Using the PBEOP, M06-2X, and B3LYP Functionals with the 6-311G(d) Basis Set for Each of the Static and Dynamic Approaches^a^

N	*E*_red,SD_	*E*_red,SC1_	*E*_red,SC2_	*E*_red,DDI_	*E*_red,DDE_	*E*_red,DMI_	*E*_red,DME_
PBEOP							
A	1.12	1.10	1.11	1.14 ± 0.22	0.17 ± 1.26	1.13 ± 0.07	1.54 ± 0.66
C	1.53	1.69	1.72	1.74 ± 0.50	1.35 ± 1.74	1.74 ± 0.28	2.27 ± 0.83
G	0.85	0.58	0.58	0.86 ± 0.24	0.29 ± 1.78	0.78 ± 0.07	1.34 ± 0.75
T	1.54	2.07	2.04	1.59 ± 0.18	0.40 ± 1.38	1.55 ± 0.07	2.08 ± 0.63
U	1.90	2.62	2.62	1.98 ± 0.22	0.76 ± 1.36	1.88 ± 0.06	2.39 ± 0.70
M06-2X							
A	1.72	1.72	1.73	1.80 ± 0.24	1.02 ± 1.41	1.72 ± 0.09	2.21 ± 0.66
C	2.41	2.15	2.50	2.20 ± 0.32	1.69 ± 1.74	2.33 ± 0.07	2.96 ± 0.73
G	1.41	1.12	1.13	1.41 ± 0.27	0.46 ± 1.75	1.37 ± 0.08	1.82 ± 0.67
T	2.05	2.64	2.64	2.08 ± 0.18	1.11 ± 1.55	2.08 ± 0.09	2.69 ± 0.69
U	2.39	3.28	3.28	2.35 ± 0.19	1.14 ± 1.45	2.41 ± 0.09	2.91 ± 0.73
B3LYP							
A	1.47	1.47	1.48	1.52 ± 0.25	0.45 ± 1.24	1.46 ± 0.07	1.85 ± 0.61
C	2.28	1.64	2.05	2.05 ± 0.28	1.36 ± 1.73	2.02 ± 0.06	2.64 ± 0.73
G	1.17	0.90	0.91	1.19 ± 0.26	2.31 ± 1.78	1.11 ± 0.09	1.28 ± 0.65
T	1.87	2.40	2.40	1.93 ± 0.16	3.50 ± 1.30	1.89 ± 0.09	2.22 ± 0.67
U	2.22	3.05	3.05	2.21 ± 0.20	3.06 ± 1.30	2.21 ± 0.07	2.54 ± 0.71

a Approaches are designated as static direct (SD), static cycle 1 (SC1), static cycle 2 (SC2), dynamic direct implicit (DDI), dynamic direct explicit (DDE), dynamic Marcus implicit (DMI), and dynamic Marcus explicit (DME). All the potentials are given in V.

If the oxidation potentials of the nucleobases are analyzed individually, PBEOP provides better results for purines (adenine and guanine) than for pyrimidines (cytosine, thymine and uracil). Actually, PBEOP slightly underestimates the potentials of adenine and guanine in all static and dynamic approaches using implicit solvation (see Table 3 and Figures 4 and 6). In the case of the dynamic direct explicit protocol, this underestimation is more important, and the potentials of purines are slightly overestimated in the dynamic Marcus explicit procedure. On the other hand, potentials for cytosine, thymine, and uracil are always overestimated by PBEOP, showing larger errors than for purines but still in good agreement with the experimental data. The only exception is found in the dynamic direct explicit approach, where the computed oxidation potentials are much lower than the experimental ones. M06-2X is the functional that presents the largest deviation from the experimental results in both static and dynamic methodologies, with the already mentioned exception of the dynamic direct explicit approach. As in the case of PBEOP, the M06-2X errors found for the purine nucleobases are lower than the errors found for the pyrimidine ones. The accuracy of the B3LYP functional is halfway between the one given by PBEOP and M06-2X. Moreover, the error of B3LYP is smaller for purines than for pyrimidines, resembling the behavior of the other two functionals.

### 4.4 Tautomerism Effects

The relative order of the one-electron oxidation potentials previously determined by experiments and theory^1,2,4,5,17,18,26,28,30,32^ is successfully reproduced by our calculations using both static and dynamic approaches: G < A < T < C < U. Thus, guanine is the most oxidizable nucleobase followed by adenine, that is, purine molecules are more prone than pyrimidines to transfer an electron to a sacrificial agent present in the environment. This can be explained in terms of the degree of electronic delocalization. Since the π system of purine derivatives is larger than the π system of pyrimidine derivatives, the energy needed to detach an electron from the molecule is smaller in purines than in pyrimidines. From a different but equivalent point of view, the positive charge generated when the cation is formed after oxidation can be more easily delocalized over the π system of purines, making the system more stable than a positive charge in pyrimidines.

Although our calculations properly reproduce the relative oxidation potentials of nucleobases, the agreement with the experimental values is not perfect. One possible source of deviation could be related to the fact that only the most stable tautomer has been considered in the different theoretical models. However, several tautomeric species can exist in protic solvents such as water, and they should be taken into account. Hobza and co-workers carried out a systematic theoretical study on the stability of the main tautomers of the nucleobases.^98−101^ Here, the relative energies of the different tautomers with respect to the canonical forms have been taken from the works by Hobza et al., and the relative population of each tautomer was calculated assuming a Boltzmann distribution. The one-electron oxidation potentials of the tautomers with a significant population (over 1%) at 298.15 K were calculated using the static direct approach. The free energy of the neutral and the cationic species of each tautomer was computed at the PBEOP/6-311G(d) level. Then, the relative populations obtained from the PBEOP/6-311G(d) energies were compared with the ones reported by Hobza and co-workers,^98−101^ which were computed at a higher lever of theory (MP2/TZVPP/PCM). The comparison shows a significant disagreement between our calculations and the ones reported in the literature, which is not surprising considering that very highly accurate energies are needed to obtain reliable populations. For instance, the relative population of the canonical adenine with respect to the second most abundant tautomer was predicted to be 57% by using the PBEOP energies, while a value of 98% was obtained with the MP2 energies. Since PBEOP was not able to correctly describe the relative populations, the relative energies between tautomers were rescaled to match the MP2 relative populations. Then, the one-electron oxidation potential was calculated as a weighted average of the reduction equilibrium constants by following the methodology suggested by Psciuk and co-workers:^2^
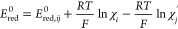
16where χ_*i*_ is the relative population of the *i*th tautomer in its oxidized form and χ′_*j*_ is the relative population of the *j*th tautomer in its reduced form.

**Figure 9:**
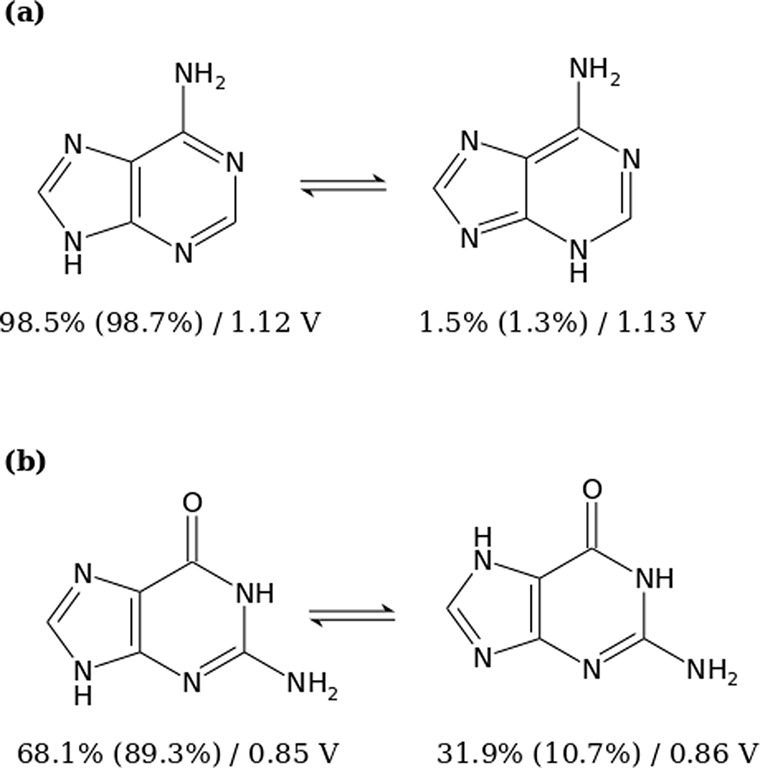
Schematic representation of the relevant tautomers that affect the value of the one-electron oxidation potentials of (a) adenine and (b) guanine. The first and second percentages below each species correspond to the relative population of the neutral and cationic forms, respectively. The one-electron oxidation potential shown for each of these molecules is associated with a situation in which there is no other tautomer in the aqueous phase. Computation of the potentials was performed by means of the static direct scheme using PBEOP/6-311G(d)/COSMO.

The relative abundance of all the tautomers of the pyrimidine nucleobases were found to be insignificant. Therefore, it can be stated that the average one-electron oxidation potentials of these molecules are completely dominated by the potential of the canonical form. In the case of adenine, the abundances of the canonical neutral and cationic species are 98.5% and 98.7%, respectively (*E*_red_ = 1.12 V), while the noncanonical forms, with a hydrogen bonded to N3 instead of N9 (see Figure 9), present an abundance of 1.5% and 1.3% for the neutral and cationic species, respectively (*E*_red_ = 1.13 V). The weighted average one-electron oxidation potential was 1.12 V. This provides evidence that the presence of a tautomer in the case of adenine can be ignored. Finally, guanine shows two important tautomers: the canonical structure with a Boltzmann population of 68.0% and 84.2% for the neutral and cationic species (*E*_red_ = 0.85 V) and a tautomer where a hydrogen migrated from N9 to N7 with a population of 31.8% and 15.8% for the neutral and cationic species (*E*_red_ = 0.86 V). The weighted average one-electron oxidation potential was 0.85 V. This means that tautomerism has no significant effect on guanine. Consequently, the study of one-electron oxidation potentials of the nucleobases can be carried out without taking into account the effect of tautomerism.

An interesting point to take into account is that the values of the one-electron oxidation potential for the two tautomers of guanine and adenine are very similar. This means that the equilibrium constants of the oxidation processes in both tautomers would be also similar. Thus, the relative population (related to the equilibrium constant of the tautomerism reaction) is going to determine the value of the average potential. Otherwise, the equilibrium constants of the oxidation processes for each tautomer should have been considered.

## 5 Conclusions

The establishment of accurate theoretical protocols to compute the redox properties of the nucleobases is of fundamental importance when modeling charge-transfer processes, such as those involved in the functioning of DNA-based biosensors. In the present work, different static and dynamic computational models using implicit and explicit solvation have been proposed and evaluated in order to compute the one-electron oxidation potentials of the five nucleobases.

Three static protocols have been employed, including the direct calculation of the oxidation potentials from the AIE (static direct) and two different thermodynamic cycles, where relaxation upon solvation is considered (static cycle 1) or ignored (static cycle 2). The static direct approach provided the most accurate results with respect to the experimental values found in the literature. Both thermodynamic cycles gave similar results for all nucleobases but cytosine, for which the static cycle 1 performed better, indicating that geometry relaxation upon solvation is relatively relevant only for this particular nucleobase.

Configurational sampling was introduced in the model by running classical MD simulations for the neutral and cationic solvated species. Then, the oxidation potential was computed directly from the energy difference between the two trajectories (dynamic direct protocol) or from the average of the VIE and VAE (dynamic Marcus protocol). The energy calculations were performed for 200 snapshots, selected from each of the trajectories, in the implicit COSMO and explicit TIP3P solvent models by polarizable-embedding QM/continuum and electrostatic-embedding QM/MM schemes. All the dynamic protocols have shown to be converged after using 100 snapshots. The application of the Marcus theory provided faster convergence than the use of the direct approach. In addition, the use of COSMO also showed better convergence behavior than the calculations carried out with TIP3P. The assessment of the Marcus regime within these systems revealed that the averaging character of the implicit solvation requires a smaller number of geometries than the explicit solvation to satisfies the Marcus theory.

The comparison of the oxidation potentials obtained by the static protocols and by the dynamic ones in implicit solvation revealed that sampling effects are not important since static and dynamic implicit calculations gave very similar results. This is particularly relevant considering that the calculation of the oxidation potential for a nucleobase by the static direct approach requires running only two geometry optimizations and two frequency calculations, while the application of a dynamic protocol requires running two classical MD simulations followed by 400 (800) single-point calculations for the direct (Marcus) approach. Therefore, the static approximations are computationally much cheaper and simpler than the dynamic ones.

The effect of using different solvation models was investigated in the dynamic framework. In general, the errors obtained in explicit solvent were larger than those obtained in implicit solvent. This demonstrated that the introduction of mutual polarization between the solute and solvent is important to obtain more accurate results. In addition, due to the nature of the COSMO model, where all possible solvent configurations are represented by the cavity in an average way in each single-point calculation, the standard deviations of the oxidation potentials along the geometries of the ensembles are much smaller in COSMO than in TIP3P. Moreover, the use of the Marcus theory improved the results obtained by the dynamic direct approach when using explicit solvent but not implicit, for which the accuracy of both dynamic protocols are very similar.

Three different functionals, namely, PBEOP, M06-2X, and B3LYP, have been employed for all static and dynamic protocols. In general, PBEOP is the functional that provided the lowest oxidation potentials and the best agreement with the experimental values for all the protocols except the dynamic direct explicit approach. In this particular case, fortuitous error cancellation makes M06-2X the best choice to describe the electronic structure of the nucleobases. Finally, the presence of different tautomers in water is only relatively relevant for adenine and guanine, but their inclusion in the theoretical model did not significantly influence the values of the one-electron oxidation potentials.

In summary, the most accurate computational protocols to compute the one-electron oxidation potentials of nucleobases in water are the static direct, dynamic direct implicit, and dynamic Marcus implicit calculations. Although the static approach seems, thus, the obvious choice due to its computational efficiency and simplicity, it is important to keep in mind that the use of dynamic approaches will likely be necessary when modeling more complex systems, such a biosensor formed by several DNA strands linked to a metal surface. In addition, the inclusion of explicit solvent molecules in the model could also be necessary if specific interactions, for example, hydrogen bonding, play an important role in such devices.

## Notes

The setup of the systems was performed with homemade scripts, the cpptraj program^102^ (https://amber-md.github.io/cpptraj/CPPTRAJ.xhtml), and the AmberTools21 package, which are available in the Amber 20 program (https://ambermd.org/).^81^ The classical MD simulations were carried out using both the CUDA version of the pmemd program and the CPU version of the sander program, available in the Amber 20 package. The QM calculations were conducted with the NWChem package (https://www.nwchem-sw.org/).^69^ Finally, the simulations were visualized with Visual Molecular Dynamics (https://www.ks.uiuc.edu/Research/vmd/).^103^

## References

[ref1] D’Annibale V.; Nardi A. N.; Amadei A.; D’Abramo M. Theoretical Characterization of the Reduction Potentials of Nucleic Acids in Solution. J. Chem. Theory Comput. 2021, 17, 1301–1307. 10.1021/acs.jctc.0c00728. 33621084 PMC8028051

[ref2] Psciuk B. T.; Lord R. L.; Munk B. H.; Schlegel H. B. Theoretical Determination of One-Electron Oxidation Potentials for Nucleic Acid Bases. J. Chem. Theory Comput. 2012, 8, 5107–5123. 10.1021/ct300550x. 26593200

[ref3] Faraggi M.; Broitman F.; Trent J. B.; Klapper M. H. One-Electron Oxidation Reactions of Some Purine and Pyrimidine Bases in Aqueous Solutions. Electrochemical and Pulse Radiolysis Studies. J. Phys. Chem. 1996, 100, 14751–14761. 10.1021/jp960590g.

[ref4] Jovanovic S. V.; Simic M. G. One-electron redox potentials of purines and pyrimidines. J. Phys. Chem. 1986, 90, 974–978. 10.1021/j100277a053.

[ref5] Seidel C. A. M.; Schulz A.; Sauer M. H. M. Nucleobase-Specific Quenching of Fluorescent Dyes. 1. Nucleobase One-Electron Redox Potentials and Their Correlation with Static and Dynamic Quenching Efficiencies. J. Phys. Chem. 1996, 100, 5541–5553. 10.1021/jp951507c.

[ref6] Ghoshdastidar D.; Ghosh D.; Senapati S. High Nucleobase-Solubilizing Ability of Low-Viscous Ionic Liquid/Water Mixtures: Measurements and Mechanism. J. Phys. Chem. B 2016, 120, 492–503. 10.1021/acs.jpcb.5b07179. 26726776

[ref7] Mehrotra P. Biosensors and their applications – A review. J. Oral. Biol. Craniofac. Res. 2016, 6, 153–159. 10.1016/j.jobcr.2015.12.002. 27195214 PMC4862100

[ref8] Kissinger P. T. Biosensors—a perspective. Biosens. Bioelectron. 2005, 20, 2512–2516. 10.1016/j.bios.2004.10.004. 15854823

[ref9] Zhai J.; Cui H.; Yang R. DNA based biosensors. Biotechnol. Adv. 1997, 15, 43–58. 10.1016/S0734-9750(97)00003-7. 14539378

[ref10] Ozkan D.; Erdem A.; Kara P.; Kerman K.; Meric B.; Hassmann J.; Ozsoz M. Allele-Specific Genotype Detection of Factor V Leiden Mutation from Polymerase Chain Reaction Amplicons Based on Label-Free Electrochemical Genosensor. Anal. Chem. 2002, 74, 5931–5936. 10.1021/ac0257905. 12498186

[ref11] Wong E. L.; Mearns F. J.; Gooding J. J. Further development of an electrochemical DNA hybridization biosensor based on long-range electron transfer. Sens. Actuators B Chem. 2005, 111–112, 515–521. 10.1016/j.snb.2005.03.072.

[ref12] Sadik O. A.; Aluoch A. O.; Zhou A. Status of biomolecular recognition using electrochemical techniques. Biosens. Bioelectron. 2009, 24, 2749–2765. 10.1016/j.bios.2008.10.003. 19054662

[ref13] Saidur M.; Aziz A. A.; Basirun W. Recent advances in DNA-based electrochemical biosensors for heavy metal ion detection: A review. Biosens. Bioelectron. 2017, 90, 125–139. 10.1016/j.bios.2016.11.039. 27886599

[ref14] Bu N.-N.; Tang C.-X.; He X.-W.; Yin X.-B. Tetrahedron-structured DNA and functional oligonucleotide for construction of an electrochemical DNA-based biosensor. Chem. Commun. 2011, 47, 7689–7691. 10.1039/c1cc11628b. 21660362

[ref15] Berlin Y. A.; Burin A. L.; Ratner M. A. DNA as a molecular wire. Superlattices Microstruct. 2000, 28, 241–252. 10.1006/spmi.2000.0915.

[ref16] Wohlgamuth C. H.; McWilliams M. A.; Slinker J. D. DNA as a Molecular Wire: Distance and Sequence Dependence. Anal. Chem. 2013, 85, 8634–8640. 10.1021/ac401229q. 23964773

[ref17] Steenken S.; Jovanovic S. V. How Easily Oxidizable Is DNA? One-Electron Reduction Potentials of Adenosine and Guanosine Radicals in Aqueous Solution. J. Am. Chem. Soc. 1997, 119, 617–618. 10.1021/ja962255b.

[ref18] Steenken S.; Jovanovic S. V.; Bietti M.; Bernhard K. The Trap Depth (in DNA) of 8-Oxo-7,8-dihydro-2‘deoxyguanosine as Derived from Electron-Transfer Equilibria in Aqueous Solution. J. Am. Chem. Soc. 2000, 122, 2373–2374. 10.1021/ja993508e.

[ref19] Burrows C. J.; Muller J. G. Oxidative Nucleobase Modifications Leading to Strand Scission. Chem. Rev. 1998, 98, 1109–1152. 10.1021/cr960421s. 11848927

[ref20] Steenken S. Purine bases, nucleosides, and nucleotides: aqueous solution redox chemistry and transformation reactions of their radical cations and e- and OH adducts. Chem. Rev. 1989, 89, 503–520. 10.1021/cr00093a003.

[ref21] Kittler L.; Löber G.; Gollmick F.; Berg H. Redox processes during photodynamic damage of DNA. J. Electroanal. Chem. Interface Electrochem. 1980, 116, 503–511. 10.1016/S0022-0728(80)80273-7.

[ref22] Reipa V.; Atha D. H.; Coskun S. H.; Sims C. M.; Nelson B. C. Controlled potential electro-oxidation of genomic DNA. PLoS One 2018, 13, e0190907 10.1371/journal.pone.0190907. 29324786 PMC5764341

[ref23] Xie H.; Yang D.; Heller A.; Gao Z. Electrocatalytic Oxidation of Guanine, Guanosine, and Guanosine Monophosphate. Biophys. J. 2007, 92, L70–L72. 10.1529/biophysj.106.102632. 17277179 PMC1831706

[ref24] Li M.-J.; Liu W.-X.; Peng C.-R.; Lu W.-C. A First-Principles Method for Predicting Redox Potentials of Nucleobases and the Metabolites in Aqueous Solution. Acta Phys.-Chim. Sin. 2011, 27, 595–603. 10.3866/PKU.WHXB20110333.

[ref25] Baik M.-H.; Silverman J. S.; Yang I. V.; Ropp P. A.; Szalai V. A.; Yang W.; Thorp H. H. Using Density Functional Theory To Design DNA Base Analogues with Low Oxidation Potentials. J. Phys. Chem. B 2001, 105, 6437–6444. 10.1021/jp010643g.

[ref26] Zhang Y.; Xie P.; Yang S.; Han K. Ionization and Electron Attachment for Nucleobases in Water. J. Phys. Chem. B 2019, 123, 1237–1247. 10.1021/acs.jpcb.8b09435. 30638023

[ref27] Thapa B.; Schlegel H. B. Calculations of pKa’s and Redox Potentials of Nucleobases with Explicit Waters and Polarizable Continuum Solvation. J. Phys. Chem. A 2015, 119, 5134–5144. 10.1021/jp5088866. 25291241

[ref28] Paukku Y.; Hill G. Theoretical Determination of One-Electron Redox Potentials for DNA Bases, Base Pairs, and Stacks. J. Phys. Chem. A 2011, 115, 4804–4810. 10.1021/jp201281t. 21500846

[ref29] Lewis K.; Copeland K.; Hill G. One-Electron Redox Properties of DNA Nucleobases and Common Tautomers. Int. J. Quantum Chem. 2014, 114, 1678–1684. 10.1002/qua.24745.

[ref30] Wang J.; Yang S.; Zhang Y. One-electron oxidation and redox potential of nucleobases and deoxyribonucleosides computed by QM/MM simulations. Chem. Phys. Lett. 2020, 739, 136948 10.1016/j.cplett.2019.136948.

[ref31] Mukherjee M.; Tripathi D.; Brehm M.; Riplinger C.; Dutta A. K. Efficient EOM-CC-based Protocol for the Calculation of Electron Affinity of Solvated Nucleobases: Uracil as a Case Study. J. Chem. Theory Comput. 2021, 17, 105–116. 10.1021/acs.jctc.0c00655. 33377775

[ref32] Crespo-Hernández C. E.; Close D. M.; Gorb L.; Leszczynski J. Determination of Redox Potentials for the Watson-Crick Base Pairs, DNA Nucleosides, and Relevant Nucleoside Analogues. J. Phys. Chem. B 2007, 111, 5386–5395. 10.1021/jp0684224. 17447808

[ref33] Adcock S. A.; McCammon J. A. Molecular Dynamics: Survey of Methods for Simulating the Activity of Proteins. Chem. Rev. 2006, 106, 1589–1615. 10.1021/cr040426m. 16683746 PMC2547409

[ref34] Braun E.; Gilmer J.; Mayes H. B.; Mobley D. L.; Monroe J. I.; Prasad S.; Zuckerman D. M. Best Practices for Foundations in Molecular Simulations [Article v1.0]. Living J. Comput. Mol. Sci. 2018, 1, 5957 10.33011/livecoms.1.1.5957. 31788666 PMC6884151

[ref35] Senn H. M.; Thiel W. QM/MM Methods for Biomolecular Systems. Angew. Chem., Int. Ed. 2009, 48, 1198–1229. 10.1002/anie.200802019. 19173328

[ref36] Jorgensen W. L.; Chandrasekhar J.; Madura J. D.; Impey R. W.; Klein M. L. Comparison of simple potential functions for simulating liquid water. J. Chem. Phys. 1983, 79, 926–935. 10.1063/1.445869.

[ref37] Aschi M.; Spezia R.; Di Nola A.; Amadei A. A first-principles method to model perturbed electronic wavefunctions: The effect of an external homogeneous electric field. Chem. Phys. Lett. 2001, 344, 374–380. 10.1016/S0009-2614(01)00638-8.

[ref38] Zanetti-Polzi L.; Del Galdo S.; Daidone I.; D’Abramo M.; Barone V.; Aschi M.; Amadei A. Extending the perturbed matrix method beyond the dipolar approximation: comparison of different levels of theory. Phys. Chem. Chem. Phys. 2018, 20, 24369–24378. 10.1039/C8CP04190C. 30215645

[ref39] Ho J. Are thermodynamic cycles necessary for continuum solvent calculation of pKas and reduction potentials?. Phys. Chem. Chem. Phys. 2015, 17, 2859–2868. 10.1039/C4CP04538F. 25503399

[ref40] Miertus S.; Scrocco E.; Tomasi J. Electrostatic interaction of a solute with a continuum. A direct utilizaion of AB initio molecular potentials for the prevision of solvent effects. Chem. Phys. 1981, 55, 117–129. 10.1016/0301-0104(81)85090-2.

[ref41] Miertus S.; Tomasi J. Approximate evaluations of the electrostatic free energy and internal energy changes in solution processes. Chem. Phys. 1982, 65, 239–245. 10.1016/0301-0104(82)85072-6.

[ref42] Pascual-ahuir J. L.; Silla E.; Tuñon I. GEPOL: An improved description of molecular surfaces. III. A new algorithm for the computation of a solvent-excluding surface. J. Comput. Chem. 1994, 15, 1127–1138. 10.1002/jcc.540151009.

[ref43] Barone V.; Cossi M.; Tomasi J. A new definition of cavities for the computation of solvation free energies by the polarizable continuum model. J. Chem. Phys. 1997, 107, 3210–3221. 10.1063/1.474671.

[ref44] Cancès E.; Mennucci B.; Tomasi J. A new integral equation formalism for the polarizable continuum model: Theoretical background and applications to isotropic and anisotropic dielectrics. J. Chem. Phys. 1997, 107, 3032–3041. 10.1063/1.474659.

[ref45] Mennucci B.; Tomasi J.; Cammi R.; Cheeseman J. R.; Frisch M. J.; Devlin F. J.; Gabriel S.; Stephens P. J. Polarizable Continuum Model (PCM) Calculations of Solvent Effects on Optical Rotations of Chiral Molecules. J. Phys. Chem. A 2002, 106, 6102–6113. 10.1021/jp020124t.

[ref46] Klamt A.; Schüürmann G. COSMO: a new approach to dielectric screening in solvents with explicit expressions for the screening energy and its gradient. J. Chem. Soc., Perkin Trans. 1993, 2, 799–805. 10.1039/P29930000799.

[ref47] York D. M.; Karplus M. A Smooth Solvation Potential Based on the Conductor-Like Screening Model. J. Phys. Chem. A 1999, 103, 11060–11079. 10.1021/jp992097l.

[ref48] Marenich A. V.; Ho J.; Coote M. L.; Cramer C. J.; Truhlar D. G. Computational electrochemistry: prediction of liquid-phase reduction potentials. Phys. Chem. Chem. Phys. 2014, 16, 15068–15106. 10.1039/C4CP01572J. 24958074

[ref49] Truhlar D. G.; Cramer C. J.; Lewis A.; Bumpus J. A. Molecular Modeling of Environmentally Important Processes: Reduction Potentials. J. Chem. Educ. 2004, 81, 596–604. 10.1021/ed081p596.

[ref50] Truhlar D. G.; Cramer C. J.; Lewis A.; Bumpus J. A. Erratum: Molecular modeling of environmentally important processes: Reduction potentials (Journal of Chemical Education 2004, 81, 596–604). J. Chem. Educ. 2007, 84, 934 10.1021/ed084p934.1.

[ref51] Isse A. A.; Gennaro A. Absolute Potential of the Standard Hydrogen Electrode and the Problem of Interconversion of Potentials in Different Solvents. J. Phys. Chem. B 2010, 114, 7894–7899. 10.1021/jp100402x. 20496903

[ref52] Kelly C. P.; Cramer C. J.; Truhlar D. G. Aqueous Solvation Free Energies of Ions and Ion-Water Clusters Based on an Accurate Value for the Absolute Aqueous Solvation Free Energy of the Proton. J. Phys. Chem. B 2006, 110, 16066–16081. 10.1021/jp063552y. 16898764

[ref53] Fornari R. P.; de Silva P. A Computational Protocol Combining DFT and Cheminformatics for Prediction of pH-Dependent Redox Potentials. Molecules 2021, 26, 3978 10.3390/molecules26133978. 34209898 PMC8271517

[ref54] Bartmess J. E. Thermodynamics of the Electron and the Proton. J. Phys. Chem. 1994, 98, 6420–6424. 10.1021/j100076a029.

[ref55] Bartmess J. E. Erratum: Thermodynamics of the Electron and the Proton (Journal of Physical Chemistry (1994) 98 (6420–6424)). J. Phys. Chem. 1995, 99, 6755 10.1021/j100017a069.

[ref56] Isse A. A.; Gennaro A. Absolute Potential of the Standard Hydrogen Electrode and the Problem of Interconversion of Potentials in Different Solvents. J. Phys. Chem. B 2010, 114, 7894–7899. 10.1021/jp100402x. 20496903

[ref57] Zwanzig R. W. High-Temperature Equation of State by a Perturbation Method. I. Nonpolar Gases. J. Chem. Phys. 1954, 22, 1420–1426. 10.1063/1.1740409.

[ref58] Blumberger J.; Tavernelli I.; Klein M. L.; Sprik M. Diabatic free energy curves and coordination fluctuations for the aqueous Ag+/Ag2+ redox couple: A biased Born-Oppenheimer molecular dynamics investigation. J. Chem. Phys. 2006, 124, 064507 10.1063/1.2162881. 16483220

[ref59] Marcus R. A. On the Theory of Oxidation-Reduction Reactions Involving Electron Transfer. I. J. Chem. Phys. 1956, 24, 966–978. 10.1063/1.1742723.

[ref60] Marcus R. A. On the Theory of Oxidation-Reduction Reactions Involving Electron Transfer. II. Applications to Data on the Rates of Isotopic Exchange Reactions. J. Chem. Phys. 1957, 26, 867–871. 10.1063/1.1743423.

[ref61] Marcus R. A. On the Theory of Oxidation-Reduction Reactions Involving Electron Transfer. III. Applications to Data on the Rates of Organic Redox Reactions. J. Chem. Phys. 1957, 26, 872–877. 10.1063/1.1743424.

[ref62] Marcus R. A. On the Theory of Oxidation-Reduction Reactions Involving Electron Transfer. V. Comparison and Properties of Electrochemical and Chemical Rate Constants. J. Phys. Chem. 1963, 67, 853–857. 10.1021/j100798a033.

[ref63] Marcus R. A. On the theory of electron-transfer reactions. VI. Unified treatment for homogeneous and electrode reactions. J. Chem. Phys. 1965, 43, 679–701. 10.1063/1.1696792.

[ref64] Marcus R. A. Electrostatic Free Energy and Other Properties of States Having Nonequilibrium Polarization. I. J. Chem. Phys. 1956, 24, 979–989. 10.1063/1.1742724.

[ref65] Cárdenas G.; Marquetand P.; Mai S.; González L. A Force Field for a Manganese-Vanadium Water Oxidation Catalyst: Redox Potentials in Solution as Showcase. Catalysts 2021, 11, 493 10.3390/catal11040493.

[ref66] Diamantis P.; Tavernelli I.; Rothlisberger U. Redox Properties of Native and Damaged DNA from Mixed Quantum Mechanical/Molecular Mechanics Molecular Dynamics Simulations. J. Chem. Theory Comput. 2020, 16, 6690–6701. 10.1021/acs.jctc.0c00568. 32926773

[ref67] Matyushov D. V.; Voth G. A. Modeling the free energy surfaces of electron transfer in condensed phases. J. Chem. Phys. 2000, 113, 5413–5424. 10.1063/1.1289886.

[ref68] Small D. W.; Matyushov D. V.; Voth G. A. The Theory of Electron Transfer Reactions: What May Be Missing?. J. Am. Chem. Soc. 2003, 125, 7470–7478. 10.1021/ja029595j. 12797822

[ref69] Valiev M.; Bylaska E. J.; Govind N.; Kowalski K.; Straatsma T. P.; Van Dam H. J. J.; Wang D.; Nieplocha J.; Apra E.; Windus T. L.; de Jong W. A. NWChem: A comprehensive and scalable open-source solution for large scale molecular simulations. Comput. Phys. Commun. 2010, 181, 1477–1489. 10.1016/j.cpc.2010.04.018.

[ref70] Perdew J. P.; Burke K.; Ernzerhof M. Generalized Gradient Approximation Made Simple. Phys. Rev. Lett. 1996, 77, 3865–3868. 10.1103/PhysRevLett.77.3865. 10062328

[ref71] Perdew J. P.; Burke K.; Ernzerhof M. Erratum: Generalized gradient gpproximation made simple (Physical Review Letters 1996, 77, 3865). Phys. Rev. Lett. 1997, 78, 1396 10.1103/PhysRevLett.78.1396. 10062328

[ref72] Tsuneda T.; Suzumura T.; Hirao K. A new one-parameter progressive Colle–Salvetti-type correlation functional. J. Chem. Phys. 1999, 110, 10664–10678. 10.1063/1.479012.

[ref73] Sarmah P.; Deka R. C. Density functional studies on the electron affinities of DNA and RNA bases. Mol. Simul. 2008, 34, 879–885. 10.1080/08927020802235664.

[ref74] Becke A. D. Density-functional thermochemistry. III. The role of exact exchange. J. Chem. Phys. 1993, 98, 5648–5652. 10.1063/1.464913.

[ref75] Lee C.; Yang W.; Parr R. G. Development of the Colle-Salvetti correlation-energy formula into a functional of the electron density. Phys. Rev. B 1988, 37, 785–789. 10.1103/PhysRevB.37.785. 9944570

[ref76] Stephens P. J.; Devlin F. J.; Chabalowski C. F.; Frisch M. J. Ab Initio Calculation of Vibrational Absorption and Circular Dichroism Spectra Using Density Functional Force Fields. J. Phys. Chem. 1994, 98, 11623–11627. 10.1021/j100096a001.

[ref77] Vosko S. H.; Wilk L.; Nusair M. Accurate spin-dependent electron liquid correlation energies for local spin density calculations: a critical analysis. Can. J. Phys. 1980, 58, 1200–1211. 10.1139/p80-159.

[ref78] Zhao Y.; Truhlar D. The M06 suite of density functionals for main group thermochemistry, thermochemical kinetics, noncovalent interactions, excited states, and transition elements: Two new functionals and systematic testing of four M06-class functionals and 12 other functionals. Theor. Chem. Acc. 2008, 120, 215–241. 10.1007/s00214-007-0310-x.

[ref79] Petersson G. A.; Bennett A.; Tensfeldt T. G.; Al-Laham M. A.; Shirley W. A.; Mantzaris J. A complete basis set model chemistry. I. The total energies of closed-shell atoms and hydrides of the first-row elements. J. Chem. Phys. 1988, 89, 2193–2218. 10.1063/1.455064.

[ref80] Petersson G. A.; Al-Laham M. A. A complete basis set model chemistry. II. Open-shell systems and the total energies of the first-row atoms. J. Chem. Phys. 1991, 94, 6081–6090. 10.1063/1.460447.

[ref81] Case D. A.; Aktulga H. M.; Belfon K.; Ben-Shalom I. Y.; Brozell S.R.; Cerutti D. S.; Cheatham T.E. III; Cisneros G. A.; Cruzeiro V. W. D.; Darden T. A.; Duke R. E.; Giambasu G.; Gilson M. K.; Gohlke H.; Goetz A. W.; Harris R.; Izadi S.; Izmailov S.A.; Jin C.; Kasavajhala K.; Kaymak M. C.; King E.; Kovalenko A.; Kurtzman T.; Lee T. S.; LeGrand S.; Li P.; Lin C.; Liu J.; Luchko T.; Luo R.; Machado M.; Man V.; Manathunga M.; Merz K. M.; Miao Y.; Mikhailovskii O.; Monard G.; Nguyen H.; O’Hearn K. A.; Onufriev A.; Pan F.; Pantano S.; Qi R.; Rahnamoun A.; Roe D.R.; Roitberg A.; Sagui C.; Schott-Verdugo S.; Shen J.; Simmerling C. L.; Skrynnikov N. R.; Smith J.; Swails J.; Walker R. C.; Wang J.; Wei H.; Wolf R. M.; Wu X.; Xue Y.; York D. M.; Zhao S.; Kollman P. A. Amber 2021; University of California, San Francisco, 2021.

[ref82] Seminario J. M. Calculation of intramolecular force fields from second-derivative tensors. Int. J. Quantum Chem. 1996, 60, 1271–1277. 10.1002/(SICI)1097-461X(1996)60:7<1271::AID-QUA8>3.0.CO;2-W.

[ref83] Wang J.; Wolf R. M.; Caldwell J. W.; Kollman P. A.; Case D. A. Development and testing of a general amber force field. J. Comput. Chem. 2004, 25, 1157–1174. 10.1002/jcc.20035. 15116359

[ref84] Joung I. S.; Cheatham T. E. Determination of Alkali and Halide Monovalent Ion Parameters for Use in Explicitly Solvated Biomolecular Simulations. J. Phys. Chem. B 2008, 112, 9020–9041. 10.1021/jp8001614. 18593145 PMC2652252

[ref85] Meza J. C. Steepest descent. Wiley Interdiscip. Rev. Comput. Stat. 2010, 2, 719–722. 10.1002/wics.117.

[ref86] Galántai A. The theory of Newton’s method. J. Comput. Appl. Math. 2000, 124, 25–44. 10.1016/S0377-0427(00)00435-0.

[ref87] Darden T.; York D.; Pedersen L. Particle Mesh Ewald: An N·log(N) Method for Ewald Sums in Large Systems. J. Chem. Phys. 1993, 98, 10089–10092. 10.1063/1.464397.

[ref88] Ryckaert J.-P.; Ciccotti G.; Berendsen H. J. Numerical integration of the cartesian equations of motion of a system with constraints: molecular dynamics of n-alkanes. J. Comput. Phys. 1977, 23, 327–341. 10.1016/0021-9991(77)90098-5.

[ref89] Hammonds K. D.; Heyes D. M. Shadow Hamiltonian in classical NVE molecular dynamics simulations: A path to long time stability. J. Chem. Phys. 2020, 152, 024114 10.1063/1.5139708. 31941339

[ref90] Yoneya M.; Berendsen H. J. C.; Hirasawa K. A Non-Iterative Matrix Method for Constraint Molecular Dynamics Simulations. Mol. Simul. 1994, 13, 395–405. 10.1080/08927029408022001.

[ref91] Nogueira J. J.; Plasser F.; González L. Electronic delocalization, charge transfer and hypochromism in the UV absorption spectrum of polyadenine unravelled by multiscale computations and quantitative wavefunction analysis. Chem. Sci. 2017, 8, 5682–5691. 10.1039/C7SC01600J. 28989607 PMC5621053

[ref92] Nogueira J. J.; González L. Computational Photophysics in the Presence of an Environment. Annu. Rev. Phys. Chem. 2018, 69, 473–497. 10.1146/annurev-physchem-050317-021013. 29490201

[ref93] Zobel J. P.; Heindl M.; Nogueira J. J.; González L. Vibrational Sampling and Solvent Effects on the Electronic Structure of the Absorption Spectrum of 2-Nitronaphthalene. J. Chem. Theory Comput. 2018, 14, 3205–3217. 10.1021/acs.jctc.8b00198. 29694042

[ref94] Zobel J. P.; Nogueira J. J.; González L. Finite-temperature Wigner phase-space sampling and temperature effects on the excited-state dynamics of 2-nitronaphthalene. Phys. Chem. Chem. Phys. 2019, 21, 13906–13915. 10.1039/C8CP03273D. 30155549

[ref95] Nogueira J. J.; Roßbach S.; Ochsenfeld C.; González L. Effect of DNA Environment on Electronically Excited States of Methylene Blue Evaluated by a Three-Layered QM/QM/MM ONIOM Scheme. J. Chem. Theory Comput. 2018, 14, 4298–4308. 10.1021/acs.jctc.8b00185. 29906110

[ref96] De Vetta M.; Menger M. F. S. J.; Nogueira J. J.; González L. Solvent Effects on Electronically Excited States: QM/Continuum Versus QM/Explicit Models. J. Phys. Chem. B 2018, 122, 2975–2984. 10.1021/acs.jpcb.7b12560. 29481750

[ref97] King G.; Warshel A. Investigation of the free energy functions for electron transfer reactions. J. Chem. Phys. 1990, 93, 8682–8692. 10.1063/1.459255.

[ref98] Hanus M.; Kabeláč M.; Rejnek J.; Ryjáček F.; Hobza P. Correlated ab Initio Study of Nucleic Acid Bases and Their Tautomers in the Gas Phase, in a Microhydrated Environment, and in Aqueous Solution. Part 3. Adenine. J. Phys. Chem. B 2004, 108, 2087–2097. 10.1021/jp036090m. 19787906

[ref99] Trygubenko S. A.; Bogdan T. V.; Rueda M.; Orozco M.; Luque F. J.; Šponer J.; Slavíček P.; Hobza P. Correlated ab initio study of nucleic acid bases and their tautomers in the gas phase, in a microhydrated environment and in aqueous solution Part 1. Cytosine. Phys. Chem. Chem. Phys. 2002, 4, 4192–4203. 10.1039/B202156K.

[ref100] Hanus M.; Ryjáček F.; Kabeláč M.; Kubař T.; Bogdan T. V.; Trygubenko S. A.; Hobza P. Correlated ab Initio Study of Nucleic Acid Bases and Their Tautomers in the Gas Phase, in a Microhydrated Environment and in Aqueous Solution. Guanine: Surprising Stabilization of Rare Tautomers in Aqueous Solution. J. Am. Chem. Soc. 2003, 125, 7678–7688. 10.1021/ja034245y. 12812509

[ref101] Rejnek J.; Hanus M.; Kabeláč M.; Ryjáček F.; Hobza P. Correlated ab initio study of nucleic acid bases and their tautomers in the gas phase, in a microhydrated environment and in aqueous solution. Part 4. Uracil and thymine. Phys. Chem. Chem. Phys. 2005, 7, 2006–2017. 10.1039/B501499A. 19787906

[ref102] Roe D. R.; Cheatham T. E. PTRAJ and CPPTRAJ: Software for Processing and Analysis of Molecular Dynamics Trajectory Data. J. Chem. Theory Comput. 2013, 9, 3084–3095. 10.1021/ct400341p. 26583988

[ref103] Humphrey W.; Dalke A.; Schulten K. VMD: Visual molecular dynamics. J. Mol. Graph. 1996, 14, 33–38. 10.1016/0263-7855(96)00018-5. 8744570

